# Decoding NADPH oxidase 4 expression in human tumors

**DOI:** 10.1016/j.redox.2017.05.016

**Published:** 2017-05-26

**Authors:** Jennifer L. Meitzler, Hala R. Makhlouf, Smitha Antony, Yongzhong Wu, Donna Butcher, Guojian Jiang, Agnes Juhasz, Jiamo Lu, Iris Dahan, Pidder Jansen-Dürr, Haymo Pircher, Ajay M. Shah, Krishnendu Roy, James H. Doroshow

**Affiliations:** a Center for Cancer Research, National Cancer Institute, NIH, Bethesda, MD 20892, USA; b Division of Cancer Treatment and Diagnosis, National Cancer Institute, NIH, Bethesda, MD 20892, USA; c Pathology/Histotechnology Laboratory, Leidos Biomedical Research, Inc., Frederick National Laboratory for Cancer Research, NIH, Frederick, MD 21702, USA; d Institute for Biomedical Aging Research and Center for Molecular Biosciences Innsbruck (CMBI), Universität Innsbruck, 6020 Innsbruck, Austria; e King's College London British Heart Foundation Centre, Cardiovascular Division, James Black Centre, London SE5 9NU, United Kingdom

**Keywords:** CCLE, Cancer cell line encyclopedia, DPI, Diphenylene iodonium, DUOX, Dual oxidase, ER, Endoplasmic reticulum, EMT, Epithelial-mesenchymal transition, H&E, Haemotoxylin and Eosin, IHC, Immunohistochemistry, NOX, NADPH oxidase, ORF, open reading frame, PI, propidium iodide, qPCR, quantitative real-time PCR, TMA, tissue microarray, TGF-β1, transforming growth factor β1, NOX4, NADPH oxidase, Monoclonal antibody, Tissue microarray, Ovarian cancer, Melanoma

## Abstract

NADPH oxidase 4 (NOX4) is a redox active, membrane-associated protein that contributes to genomic instability, redox signaling, and radiation sensitivity in human cancers based on its capacity to generate H_2_O_2_ constitutively. Most studies of NOX4 in malignancy have focused on the evaluation of a small number of tumor cell lines and not on human tumor specimens themselves; furthermore, these studies have often employed immunological tools that have not been well characterized. To determine the prevalence of NOX4 expression across a broad range of solid tumors, we developed a novel monoclonal antibody that recognizes a specific extracellular region of the human NOX4 protein, and that does not cross-react with any of the other six members of the NOX gene family. Evaluation of 20 sets of epithelial tumors revealed, for the first time, high levels of NOX4 expression in carcinomas of the head and neck (15/19 patients), esophagus (12/18 patients), bladder (10/19 patients), ovary (6/17 patients), and prostate (7/19 patients), as well as malignant melanoma (7/15 patients) when these tumors were compared to histologically-uninvolved specimens from the same organs. Detection of NOX4 protein upregulation by low levels of TGF-β1 demonstrated the sensitivity of this new probe; and immunofluorescence experiments found that high levels of endogenous NOX4 expression in ovarian cancer cells were only demonstrable associated with perinuclear membranes. These studies suggest that NOX4 expression is upregulated, compared to normal tissues, in a well-defined, and specific group of human carcinomas, and that its expression is localized on intracellular membranes in a fashion that could modulate oxidative DNA damage.

## Highlights

• NADPH oxidase 4 (NOX4) detection has been achieved with a monoclonal antibody (47-6) developed to the extracellular E-loop region (amino acids 209–282).
• Tissue microarray analysis of 20 human malignancies reveals differential NOX4 expression by IHC was higher in bladder, esophageal, head and neck, ovary, and prostate cancers and melanoma than in normal tissues.
• NOX4 expression in the ovarian cancer cell line COV362 is localized in the perinuclear region.

## Graphical abstract

**Figure:**
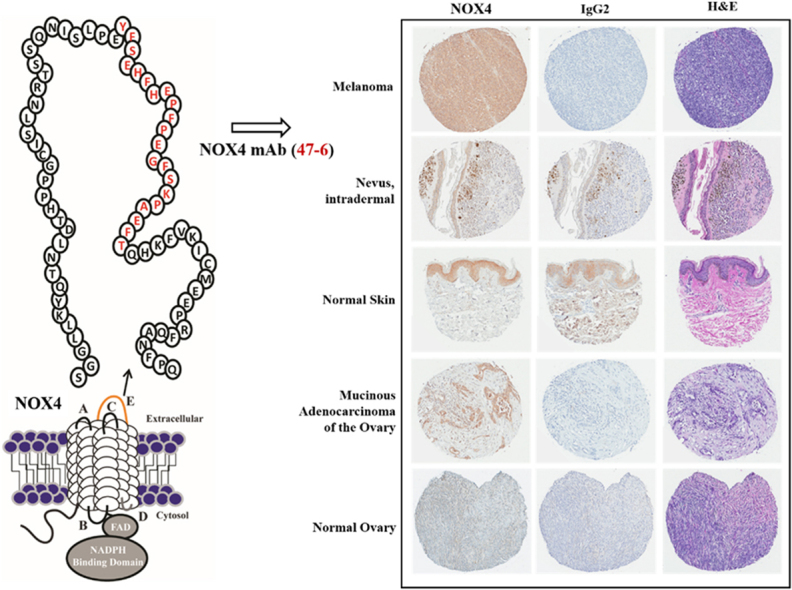

## 1 Introduction

Cellular redox balance relies on a dynamic interplay between endogenous [mitochondria, cytochrome P-450, nitric oxide synthase (NOS) and NADPH oxidase (NOX) enzymes] and exogenous (environmental agents, pharmaceuticals, and industrial chemicals) production of reactive oxygen species (ROS) and a broad array of intrinsic cellular antioxidant pathways [1], [2], [3]. Understanding the mechanisms by which ROS imbalance results in epithelial dysfunction and promotes tumor growth and progression could focus development of new redox-based strategies for therapeutic intervention. The NOX enzymatic family members (NOX1-5, DUOX1-2), through isoform specific superoxide or hydrogen peroxide production, have been associated with tissue remodeling, resistance to apoptosis, tumor cell proliferation and metastasis, and enhanced angiogenesis. Tumor promotion by NOX isoforms occurs, in part, by inflammation- and hypoxia-mediated upregulation of oxidative DNA damage and tissue injury [4], [5], [6], [7], [8].

The relationship between NOX-related oxidant production and cancer is both NOX-isoform and tumor context specific. For example, aberrant hypermethylation of the DUOX1 and DUOX2 promoters results in downregulation of both DUOXs in lung cancer [9]. DUOX1 has also been implicated in the process of wound repair [10]; recently, loss of DUOX1 expression has been linked to a loss of E-cadherin and subsequent enhanced epithelial-mesenchymal transition (EMT) [11]. DUOX2 expression, unlike that of the DUOX1 homolog, is associated with progression of pancreatic cancer; the pro-inflammatory cytokine IFN-γ triggers Stat1-mediated DUOX2/DUOXA2 up-regulation in pancreatic cancer cell lines, contributing to increased intra- and extracellular ROS production [12]. Increased DUOX2-derived ROS production is also associated with increased VEGF and HIF-1α expression in pancreatic cancer cell lines; evaluation of malignant versus matched non-malignant pancreatic tissues also demonstrated DUOX2-related up-regulation of VEGF expression [13]. While DUOX2 has also been implicated in other gastrointestinal malignancies, NOX1 is primarily involved in the proliferative potential of colonic malignancies. Profiles of pre-cancerous large bowel adenomas, as well as moderately- and well-differentiated adenocarcinomas of the colon, have demonstrated substantial NOX1 overexpression [7], [14]. It has been proposed that increased ROS levels derived from elevated NOX1 expression may stimulate tumor initiation through activation of NF-κB signaling [15], [16].

Studies of NOX4 in cancer have focused on malignancies of the brain, breast, bladder, liver, kidney, and ovary, as well as melanoma [17], [18], [19], [20], [21], [22], [23], primarily in human tumor cell lines. NOX4 has been associated with the formation of invadopodia [24], cell proliferation [25], differentiation [26], and EMT [20]. A membrane-bound, hydrogen peroxide-producing enzyme, NOX4 requires a single interaction partner for its activity, p22^ph^^o^^x^ [27], [28], [29], [30], [31]. ROS production by this isoform is constitutive, in contrast to the other six NOX family members, whose activities are regulated by protein partner translocation or calcium stimulation [30], [32], [33]. Constitutive activity is unexpected in view of the harmful effects of excessive ROS production; but, to date, enzyme abundance, stimulated by TGF-β1 [25], [34], [35], [36], [37], hypoxia and hyperoxia [38], [39], [40], [41], or vascular injury [42], [43], appears to be the only known mechanism controlling NOX4 ROS production. Furthermore, NOX4 is widely distributed; its expression has been demonstrated in osteoclasts [44], fibroblasts [34], [37], and adipocytes [45], as well as endothelial [46] and mesangial cells [47]. Developing a unified understanding of the cellular localization and physiological function of NOX4 remains an area of active investigation.

Conflicting reports place NOX4 on the membranes of perinuclear vesicles [30], in the nucleus [48], [49], [50], mitochondria [51], or endoplasmic reticulum (ER) [29], [30], [33], [52], in focal adhesions [50] or on the plasma membrane [53], [54]. These reported differences may be the result of cell type, overexpression system design (i.e. affinity tag placement), and/or specificity of the antibody used for study. Approximately 40 commercial NOX4 antibodies are currently available; most are polyclonal and rabbit-derived. Academic antibody sources have generally proven to be the only reliable NOX4 antibodies to date, but most lack monoclonal character and/or were raised against an intracellular antigen [55]. The lack of freely available, well-characterized, sensitive antibodies for investigative efforts has limited progress evaluating endogenous expression, localization, and tissue distribution. Here, we present comprehensive characterization of a novel NOX4 monoclonal antibody raised to an extracellular domain, and demonstrate its utility for investigating the role of NOX4 in tumor biology. Evaluation of antibody binding to portions of the E-loop region defined a recognition motif of 21 amino acids; sequence divergence with mouse Nox4 suggested distinct detection of the human NOX4 protein, which was experimentally verified for our monoclonal antibody. Through application of this new tool, we found that TGF-β1-stimulated fibroblasts demonstrated sensitivity to a lower, biologically relevant level of NOX4 protein. Of greatest pertinence, extensive screening of a panel of 20 malignancies and associated normal tissues investigated the relative expression of NOX4 in human epithelial tumors. Defined differential NOX4 expression favoring increased protein in tumor was found for carcinomas of the bladder, esophagus, head and neck, prostate, and ovary as well as malignant melanoma. Importantly, NOX4 was expressed in almost all of the melanomas studied (14/15, 93%) and none of the normal skin samples (n=8); overexpression of NOX4 was detected in 15/17 (88%) of ovarian carcinomas. Finally, subsequent investigations in ovarian cancer cell lines established that NOX4 protein is localized to perinuclear membranes.

## 2 Materials and methods

### 2.1 Materials, facilities, and general instrumentation

Recombinant human TGF-β1 (catalog no. 7754-BH-005) was purchased from R&D Systems. Anti-p22^ph^°^x^ antibody (catalog no. FL-195), NOX2 antibody 54.1 (catalog no. sc-130543) and Rabbit IgG (catalog no. sc-2027) were purchased from Santa Cruz Biotechnology. Anti-β-actin (catalog no. 3700), anti-HA-tag (catalog no. 2367), anti-Myc-tag (catalog no. 2276), anti-GAPDH (catalog no. 2118), anti-Vinculin (catalog no. 13901), anti-Na/K-ATPase (catalog no. 3010) and the SMAD 2/3 antibody kit (catalog no. 12747) were purchased from Cell Signaling Technology. The NOX1 mouse monoclonal antibody (NOX1-Hyb-Clone-22) used for Western analysis was raised against a truncated recombinant protein representing 341 amino acids (224–564 amino acid sequence) at the carboxyl-terminus of the human NOX1 protein [56]. DUOX protein immunoblot detection was performed using a mouse monoclonal antibody S-40 that was raised against the human DUOX2 131–540 amino acid fragment [57]. DNA sequencing was performed by Genewiz (Frederick, MD). The entire gene insert was completely sequenced for each plasmid construct. All experiments were performed at room temperature unless otherwise stated. Results are expressed as the mean ± S.D. from at least triplicate experiments. Statistical differences between mean values of control and treated samples were assessed using Student's *t*-test; p < 0.05 was considered statistically significant. Significance levels were designated as *, *p* < 0.05 and ***, *p* < 0.001 throughout.

### 2.2 Generation of the rabbit monoclonal NOX4 antibody

Immunization of rabbits and NOX4 monoclonal antibody production were carried out by Abcam, (Burlingame, CA) using the following procedure. Overexpressing NOX4 HEK293 stable cells were harvested from culture plates (500 million cells) and ethanol fixed in 100 million cell aliquots, subsequently provided to Abcam. A second 74 amino acid peptide immunogen was synthesized (NOX4 amino acids 209–282) representing the extracellular E-loop region of the human NOX4 protein. After six alternating rounds of immunization with fixed cells or peptide immunogen, the harvested serum titer reached significance as tested by ELISA against the immunogenic peptide. Subsequent to hybridoma fusion, supernatants were collected and multi-clones were evaluated for antigenic response. Six multiclones were selected and subcloned; supernatants harvested from 3 subclones (developed from each multiclone) were received and evaluated. One subclone from each multi-clone was chosen for antibody purification. After extensive evaluation, subclone 47-6 was chosen for sequencing and exclusive use in NOX4 studies.

### 2.3 Sequencing of the variable region of the NOX4 rabbit mAb coding region (GenScript)

Total RNA was extracted from the NOX4 hybridoma clone 47-6 using TRIzol reagent and analyzed by gel electrophoresis. RT-PCR was performed using isotype-specific antisense primers or universal primers according to the technical manual of the PrimeScript First Strand cDNA Synthesis Kit (catalog no. 6110 A, Clontech). Amplified antibody fragments were separately cloned into a standard cloning vector using standard molecular cloning procedures. Colony PCR screening was performed to identify clones with inserts of correct sizes. Five single colonies with inserts of correct sizes were sequenced for each antibody fragment (V_H_ and V_L_).

### 2.4 Cell culture and transfection

HEK293 (CRL-1573) embryonic kidney and CCD-19Lu (CCL-210) lung fibroblast cells were obtained from ATCC (Manassas, VA) and cultured using ATCC recommended medium supplemented with 10% FBS. COV362 ovarian cancer cells were obtained from Sigma Aldrich (catalog no. 07071910) and cultured using DMEM medium supplemented with 10% FBS. SKOV3 ovarian cancer cells and RPMI 8226 myeloma cells were obtained from the Developmental Therapeutics Program of the National Cancer Institute (Frederick National Laboratory, Frederick, MD) and cultured in McCoy's 5 A medium supplemented with 10% FBS and RPMI-1640 medium supplemented with 10% FBS, respectively. Each cell line identity was confirmed by the Genetic Resources Core Facility of Johns Hopkins University (Baltimore, MD, USA). All cell lines were tested to ensure the absence of *Mycoplasma* contamination and maintained at 37 °C in a humidified atmosphere of 5% CO_2_ and 95% air. cDNA transfection into cells was carried out using the Amaxa Nucleofector™ system from Lonza, according to the manufacturer's protocol.

For transient transfections of plasmid DNA, [pCMV-MycDDK-HsNOX4 (catalog no. RC208007, Origene) or pCMV-MmNOX4-3xHA6His (EX-Mm06833-M08, GeneCopoeia)] 4 μg cDNA was transfected into HEK293 using the Lonza system (Kit V, Program Q-001). Cells were incubated for 48 h at 37 °C before harvest and evaluation. To generate a stable, clonal cell line overexpressing NOX4, HEK293 cells were transfected with a pCMV-MycDDK-HsNOX4 plasmid or pCMV-Entry vector again using the Lonza system (Kit V, Program Q-001). Resistant clones were selected with 750 µg/mL G418 (catalog no. 5005; Teknova, Hollister, CA), and single clones were then maintained under G418 selection. For antibody selectivity studies, both NOX1- and NOX5-overexpressing cell lines were developed in-house. Briefly, stable NOX1/NOXA1/NOXO1 cells were initiated by transfection of pCMV-NOX1(3 μg) plasmid in HEK293 cells using the Lonza system (Kit V, Program Q-001), followed by selection with G418. After stable clones were achieved and validated by qPCR, a single clone was selected for transfection with pCMV-NOXA1/NOXO1 (3 μg) and single clones were selected with puromycin. The final, active NOX1 overexpression clonal cell line was maintained with 500 μg/mL G418 and 500 ng/mL puromycin (catalog no. P9620, Sigma). To generate human cells that stably over-express NOX5, KARPAS 299 lymphoma cells (Sigma) were transfected with pcDNA3-NOX5β plasmid (a kind gift from Dr. David J. R. Fulton, Medical College of Georgia) using lipofectamine2000 (Invitrogen, Carlsbad, CA, USA) per the manufacturer's protocol. Resistant clones were selected with 800 μg/mL G418, and then single clones were selected and maintained in RPMI-1640 media supplemented with 2 mM glutamine and 10% FBS, under 500 µg/mL G418 selection. KARPAS 299-NOX5 overexpressing clones were validated by Western analysis using antibodies specific for NOX5 protein and the HA tag [58]. The stable HEK293 cell line expressing both the human DUOX2 and DUOXA2 enzymes was kindly provided by Dr. William M. Nauseef (University of Iowa, Iowa City, IA, USA) and maintained in DMEM:F12 medium supplemented with 10% FBS, 800 μg/mL G418 and 250 μg/mL Zeocin (catalog no. 46–0509; Invitrogen).

### 2.5 RNA isolation and quantitative real-time PCR analysis

For quantitative real-time PCR (qPCR), total RNA was extracted from cells using the RNeasy Mini Kit (catalog no. 74104; Qiagen) according to the manufacturer's instructions. Following isolation, RNA concentrations and purity were measured on the Nanodrop ND-1000 apparatus (Nanodrop Technologies, Wilmington, DE). Two μg of total RNA was used for cDNA synthesis, and combined with SuperScript II reverse transcriptase (catalog no. 100004925; Invitrogen) and random primers (catalog no. 48190-011; Invitrogen) in a 20 μL reaction system. Cycling conditions were as follows: 25 °C for 10 min, 42 °C for 50 min, 70 °C for 15 min. After reaction, the products were diluted with H_2_O to 100 μL and RT-PCR was performed on 384-well plates in a 20 μL reaction system containing 2 μL of diluted cDNA and 1 μL of appropriate primer. Human NOX4 primer (catalog no. Hs00418356_m1), mouse NOX4 primer (catalog no. Mm00479246_m1), human CYBA (p22^phox^, catalog no. Hs00164370_m1), human β-actin (catalog no. Hs99999903_m1), and TaqMan Universal PCR mix (catalog no. 4364340) were purchased from Applied Biosystems for the reaction. PCR amplification was performed on an ABI 7900HT sequence detection system (Applied Biosystems, Foster City, CA). Relative gene expression was calculated as the ratio of the target gene to the internal reference gene (β-actin) multiplied by 10^6^ based on the C_t_ values.

### 2.6 Plasmid constructs for antibody recognition site identification

Mutations encoding stop codons in pCMV-MycDDK-HsNOX4 were introduced by QuikChange site-directed mutagenesis (Clontech), according to the manufacturer's instructions, to create four truncated NOX4 constructs. Primer sequences are listed in Supplementary Table 1; (pJLM123-126). The entire open reading frame was sequenced for each new plasmid.

### 2.7 Western analysis

For preparation of whole-cell extracts, cell pellets from HEK293, NOX4 HEK293 stable clones, and CCL-210 cells were lysed with 1X RIPA lysis buffer (catalog no. 20–188; Millipore, Temecula, CA), with the addition of a 1X protease inhibitor cocktail (catalog no. 11–836–153001; Roche) and 1 mM PMSF. The protein concentrations of resulting lysates were measured by using the BCA Protein Assay Kit (Pierce). Cell extracts were mixed with an equal volume of 2X SDS protein gel loading buffer (catalog no. 351-082-661; Quality Biological) and loaded, without boiling, onto a 4–20% Tris glycine gel (catalog no. EC6028; Invitrogen). Proteins were separated and dry transferred to nitro-cellulose membranes using the iBlot 2 gel transfer system (Thermo Fisher Scientific). The membranes were blocked in 1X TBST buffer with 5% non-fat milk or 1X TBS buffer with 3% non-fat milk for 1 h at room temperature and then incubated with primary antibody overnight in TBST (milk) buffer. Membranes were washed three times in 1X TBS buffer and incubated with HRP-conjugated secondary antibody for 1–2 h at room temperature with shaking. The antigen-antibody complex was visualized with SuperSignal West Pico Luminol/Enhancer Solution (catalog no. 1856136, Thermo Fisher Scientific) or ECL-Plus reagent (catalog no. 32132; Pierce).

### 2.8 Membrane preparation of COV362 and OVCAR3 cell lines

Approximately 200 million cells were harvested for both COV362 and OVCAR3 cell lines, washed with PBS, and stored as frozen pellets at −80 °C until ready for preparation. On the day of preparation, cell pellets were thawed on ice, then resuspended in 1.25 mL sonication buffer (11% sucrose, 120 mM NaCl, 1 mM EGTA in PBS, pH 7.4 + 1 mM PMSF added just prior to sonication). The cell suspensions were sonicated (2X) for lysis and the resulting lysates were centrifuged to collect unbroken cells and nuclei (200g, 10 min, 4 °C). The cell pellets were resuspended in 500 μL sonication buffer after which sonication (1X) and centrifugation were repeated. Both lysates collected from above the cell pellets were combined for application to a sucrose gradient. A discontinuous sucrose gradient was prepared at 4 °C, consisting of 17% and 40% (w/v) sucrose layers. The lysates were carefully added to the top of the gradient and centrifuged at 40,000 rpm (Beckman XL-90 ultracentrifuge) for 30 min. Fractions were isolated from the top of the gradient to the 17%/40% sucrose layer interface in 1 mL increments; cytosolic protein was collected as the second fraction within the 17% layer, and membrane protein was isolated as fraction 8 at the sucrose layer interface.

### 2.9 siRNA-mediated NOX4 gene silencing

COV362 cells were transfected with 5 nM non-targeting pool siRNA (catalog no. D-001810-10-05), NOX4 SMARTpool siRNA (catalog no. L-010194-00), or NOX4 #07 individual siRNA (catalog no. J-010194-07) from Dharmacon, utilizing RNAiMAX Lipofectamine™ transfection reagent (catalog no. 13778; Invitrogen). Transfected cells were harvested 96 h post-treatment, and silencing efficiency was evaluated at both the RNA (qPCR) and protein level (immunoblot).

### 2.10 Extracellular H_2_O_2_ measurement using Amplex Red®

The Amplex Red® Hydrogen Peroxide/Peroxidase Assay Kit (catalog no. A22188; Invitrogen) was used to detect extracellular H_2_O_2_ release. Assay kit buffer was replaced with KRPG buffer to facilitate whole cell assays of significant time duration (up to 2 h). The fluorescence of the oxidized 10-acetyl-3,7-dihydroxyphenoxazine was measured at an excitation wavelength of 530 nm and an emission wavelength of 590 nm, using a SpectraMax Multiplate reader (Molecular Devices, Sunnyvale, CA); 20,000 cells per well were evaluated for each assay condition, in triplicate·H_2_O_2_ was quantified by an H_2_O_2_ standard curve over a concentration range from 0 to 2 μM.

### 2.11 Superoxide anion assay

Superoxide production was measured using chemiluminescent probe L-012. As a positive control, a single site mutation (H222Q), reported to result in superoxide production, was generated in pCMV-MycDDK-HsNOX4 using Quikchange mutagenesis. Briefly, HBTS buffer was prepared prior to cell trypsinization. HEK293 parental, H222Q mutant NOX4 and wild-type NOX4 stable cell lines were counted after detachment and washed with assay buffer once; 750,000 cells per well were resuspended in HBTS for assay plate addition. A 20 μM reaction solution of L-012 in HBTS was prepared (with or without antibody or SOD); 100 μL reaction solution was combined with 100 μL resuspended cells per well and the resulting assay was monitored by chemiluminescence at 37 °C for 30 min.

### 2.12 Immunofluorescence detection by confocal microscopy

Parental and stable NOX4 overexpressing HEK293 cells (Clone WT1 and WT2) were visualized using Permanox 4-well chamber slides (catalog no. 177437; Thermo Fisher Scientific). Prior to cell addition, chamber slides were treated with a sterile solution of Poly-d-lysine (catalog no. P7405; Sigma Aldrich) for 5 min. Slides were allowed to dry for at least 2 h after solution removal prior to seeding. HEK293 parental and NOX4 overexpressing cells (5.0×10^3^) were fixed 72 h post seeding, with 4% (w/v) paraformaldehyde for 10 min at room temperature. After two washes with PBS, cells left non-permeabilized were blocked with 4% BSA in PBS at room temperature for one hour. For permeabilization, cells were treated with 0.1% Triton X-100 (v/v) for 5 min prior to blocking. After one remaining wash, the cells were incubated overnight at 4 °C with the rabbit monoclonal NOX4 (47-6) antibody at 2 μg/mL in PBS containing 0.5% (w/v) BSA. As a negative control, select cells were incubated with rabbit IgG. Following 3 washes with PBS, a secondary Alexa Fluor 488 goat anti-rabbit antibody [1:1000 in PBS containing 0.5% (w/v) BSA], catalog no. A-11034 was added. After a 1 h incubation, cells were washed, and the cell nuclei were stained with propidium iodide (PI, 0.5 µg/mL). Samples were then mounted in Vectashield solution, sealed, and stored at room temperature in the dark.

COV362 and SKOV3 ovarian cancer cells were visualized using glass 4-well chamber slides (catalog no. 154526; Thermo Fisher Scientific). The slides were pretreated with Poly-d-lysine, as previously described for Permanox slides, prior to cell addition. COV362 (2.5×10^3^) and SKOV3 (2.0×10^3^) cells were attached and fixed 72 h post seeding. Fixation and antibody staining proceeded as previously described, modified after secondary antibody treatment with rhodamine phalloidin staining (catalog no. R415; Molecular Probes), 25 μL/1 mL PBS for 10 min at room temperature, followed by mounting with Vectashield containing DAPI.

Confocal microscopy was carried out at the confocal microscopy core facility (NCI) using a Zeiss LSM 710 NLO confocal scanning microscope equipped with an Argon/2 laser (458, 488, and 514 nm), a diode laser (561 nm), and a HeNe laser (633 nm).

### 2.13 Immunodetection by flow cytometry

Log phase parental, vector control, and stable NOX4-overexpressing (Clone WT1 and WT2) HEK293 cells were trypsinized, washed in cold PBS, pelleted, and resuspended in a cold PBS/FBS solution (PBS containing 5% fetal bovine serum). Live cells were not fixed prior to labeling to promote extracellular epitope recognition. Cells to be permeablized were centrifuged for 5 min at 2000 rpm and fixed with cold 2% (v/v) paraformaldehyde for 30 min on ice. Following centrifugation, the cell pellets were resuspended in room temperature Tween 20 solution (0.15% in PBS) for 15 min in a 37 °C water bath. After centrifugation, cells (10^6^) were either unlabeled and resuspended in PBS or labeled with irrelevant rabbit IgG antibody (4 µg) or NOX4-specific rabbit monoclonal antibody (47-6, 4 µg) in human AB serum (heat-inactivated) for 30 min at 4 °C. The labeled cells were then washed twice with Tween 20 solution (0.15% in PBS) followed by incubation with secondary Alexa Fluor 488 goat anti-rabbit antibody [diluted 1:1000 in human AB serum (heat-inactivated)] for 20 min on ice in the dark. Cells were then centrifuged, washed and resuspended in PBS. Fluorescence intensity of the cells was measured on a FACScalibur (Becton Dickinson Biosciences, San Jose, CA) cytometer, acquired using the data file acquisition program CellQuest (Becton Dickinson Biosciences, San Jose, CA) and analyzed using the FlowJo software.

### 2.14 Immunohistochemistry and tissue microarray

Pellets of SKOV3, COV362, HEK293 vector control, and overexpressing NOX4 (WT1) cells were fixed in 10% buffered neutral formalin and used as controls to optimize conditions for NOX4 immunohistochemistry (IHC). Optimal conditions were achieved for the NOX4 mAb (47-6) at a dilution of 1:750 after heat-induced epitope retrieval in citrate buffer (BioGenex) in a decloaking chamber (Biocare). Primary antibody incubation occurred overnight at 4 °C, followed by biotinylated secondary goat anti-rabbit IgG (Vector Laboratories), Vectastain ABC Elite (Vector Labs) and DAB chromagen. All samples were processed in parallel with a no-primary-antibody control to evaluate possible artifactual nonspecific staining from the secondary antibody. Isotype control staining was prepared with normal rabbit IgG (Cell Signaling Technology) at a comparable concentration to the primary antibody. Following established conditions from the control samples, IHC was performed on a BioMax multiple organ tissue microarray (TMA) MC6163. The TMA was deparaffinized in alcohol and rehydrated with graded alcohol prior to antigen retrieval. After immunostaining with the optimized protocol, slide(s) were bleached with hydrogen peroxide/potassium hydroxide for 60 min at 37 °C followed by a 20 s acetic acid rinse to eliminate melanin pigment. Post bleaching, slide(s) were counterstained with hematoxylin, dehydrated, and cover slipped. Each slide was digitally imaged using an Aperio ScanScope®. The verification of staining performance was confirmed on a series of cancer tissue samples. In addition, a series of normal, nontumor tissues were evaluated to establish immunoreactivity and assay specificity. Positive controls that were expected to demonstrate different levels of NOX4 included formalin-fixed paraffin embedded human liver. Evaluation of staining on sections exposed to the primary and secondary antibodies was compared to negative control sections that were not exposed to the primary antibody. Tissues were scored as positively stained only if they exhibited a staining pattern with the primary antibody that was significantly different than that found by omitting the primary antibody. Those that did not demonstrate a significant difference between staining with and without the primary antibody were graded as 0+ (no stain), and 0% stained. Tissues that demonstrated a significant difference between the two conditions were graded as described below. The assay was interpreted with a scoring system of 0+, 1+, 2+ and 3+, for staining intensity corresponding to negative, weak, moderate, and strong NOX4 staining. The percentage of stained tumor/lesion cells (distribution) was estimated for each case, where 0% to < 10% were considered negative.

## 3 Results

### 3.1 Monoclonal antibody development targeting the extracellular NOX4 E-loop region

**Fig. 1:**
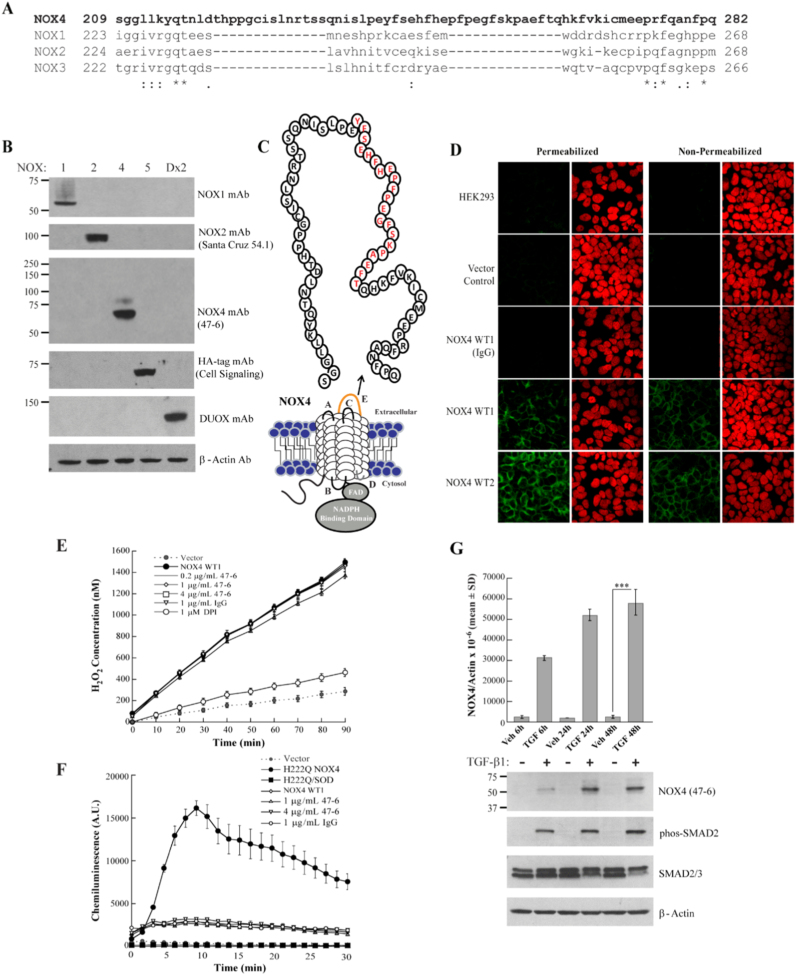
Comprehensive characterization of NOX4 monoclonal antibody 47-6. (A) Multiple sequence alignment performed using Clustal Omega of the E-loop region residues for the NOX1-4 enzymes. NOX5 and DUOX1/2 have short E-loop regions (< 10 amino acids) and therefore were not amenable to sequence alignment. (B) Western analysis carried out with samples positive for NOX1 (64.9 kDa, HEK293 NOX1, NOXA1/NOXO1 stable cell line), NOX2 (~100 kDa, glycosylated form, RPMI 8226 human myeloma cell line), NOX4 (69.7 kDa, HEK293 MycDDK-NOX4 stable cell line WT1), NOX5 (83.1 kDa, KARPAS 299 HA-NOX5 stable cell line), and DUOX2 (Dx2) (175.4 kDa, HEK293 DUOX2/DUOXA2 stable cell line) expression. Twenty micrograms per lane of total protein were loaded. Antibodies used to detect the various NADPH homologues are listed, with details in the Methods section. (C) Schematic illustration of the NOX4 E-loop; the validated recognition sequence is highlighted (red). (D) Confocal microscopy of HEK293 parental, vector control, and MycDDK-NOX4 stable clones 1 (WT1) and 2 (WT2) under permeabilized and non-permeabilized conditions. Cells were incubated with 2 μg/mL 47-6 NOX4 mAb or rabbit IgG (control) prior to secondary antibody staining with Alexa Fluor 488 (Green) and PI nuclei staining (red). (E) H_2_O_2_ production by HEK293 NOX4 stable clone 1 versus stable vector control cells measured with the Amplex Red assay. NOX4 overexpressing stable cells were pre-incubated for 30 min at 37 °C with 47-6 mAb, rabbit IgG or DPI before addition to Amplex reagent solution. The assay was monitored every 10 min for 90 min. (F) L-012 Assay measured superoxide generation across superoxide producing stable mutant H222Q NOX4 and WT NOX4 overexpressing stable cells treated with 47-6 mAb or rabbit IgG. SOD (4U) treatment was used to verify superoxide production over 30 min, data was collected every 1.5 min for 30 min. (G) TGF-β1-stimulated NOX4 up-regulation in lung fibroblast cells verified by RNA quantification and immunoblot analysis. Top: Quantitative real-time RT-PCR assay of relative NOX4 expression from 24 h starved lung fibroblasts (CCL-210) treated with vehicle control (Veh) or TGF-β1 (TGF) for 6 h, 24 h, or 48 h, normalized to β-Actin. This data represents the mean and standard deviation from triplicate samples. Bottom: Western analysis of whole cell lysates from lung fibroblasts treated with TGF-β1; 80 μg total protein loaded per lane. TGF-β1 treatment was verified by the detection of phosphorylated SMAD2. ***, *p* < 0.001. (For interpretation of the references to color in this figure legend, the reader is referred to the web version of this article.)

Generation of a monoclonal antibody to the extracellular domain of NOX4 with sufficient sensitivity and specificity to monitor changes in enzyme expression by Western blot and TMA for levels of cancer expression has proven difficult. Many commercial antibodies have failed in studies attempting to conclusively establish NOX4 localization and validate the knockdown of NOX4 expression [59], [60]. Our approach toward development of an effective antibody focused on an extracellular region of the NOX4 enzyme termed the E-loop (Fig. 1). Single site mutation studies within this domain suggest that its disulfide-supported secondary structure or metal binding capability may facilitate superoxide to hydrogen peroxide conversion [61]. Targeting this region could therefore afford some control over ROS generation for therapeutic purposes. A peptide comprised of the amino acids predicted to be found in this exposed cellular region (amino acids 209–282) was synthesized. A second immunogen was prepared to permit alternating rounds of immunization using fixed aliquots of stable NOX4-overexpressing HEK293 cell membranes. While the NOX4 amino acid sequence does not display ER retention or endosomal translocation signals [62], previous studies have reported a lack of NOX4 enzyme at the plasma membrane surface in overexpression systems established in HEK293, COS-7, or HeLa parental cell lines [29], [30], [33], [62]. Recently, plasma membrane association was reported in H661 and COS-7 cells [53], [63]; lack of recognition in earlier studies may be the result of the detection system utilized or disruption of trafficking due to construct design. Plasma membrane purification was performed, and surface localization confirmed in our overexpressing cells employed as a secondary antigen (data not shown). Six alternating injections were performed on 2 rabbit hosts, resulting in 30 ELISA-positive multiclones. Of these, 6 hybridoma multiclones were selected by Western analysis for subcloning (Supplementary Fig. 1). One subclone from each multiclone (after initial Western blot analysis) was purified by peptide affinity column for activity studies; finally, subclone 47-6 was chosen for sequencing (Supplementary Fig. 2) and subsequent use in this study.

### 3.2 Determination of NOX4 antibody (47-6) specificity by Western analysis

The NOX4 protein has been identified in a remarkable number of cell lines, tissues, and animal model systems, unfortunately, often without routine RNA validation. Many systems which may contain the NOX4 protein also exhibit expression of other NADPH oxidases. Sequence identity is shared between NOX4 and its NOX family members as follows: NOX1 (36%), NOX2 (39%), NOX3 (36%), and NOX5 (27%). Interestingly, the NOX4 E-loop is significantly longer than those found in other NOXs (Fig. 1A); this difference in length has been conjectured to contribute to ROS species conversion [61], [64]. To ascertain whether our NOX4 antibody is capable of distinguishing between known NOX isoforms, specific, high expression systems for each oxidase were established and compared at the protein level (Fig. 1B). The NOX4 monoclonal antibody detected an intense band at ~65 kDa from total protein isolated from stable NOX4 overexpressing cells, consistent with the calculated molecular weight. No cross reactivity with any other NOX protein was observed, nor were any significant non-specific bands detected. A low intensity higher molecular weight band (~80 kDa) was also observed for NOX4, and is consistent with previous observations in HeLa, HUVEC, and vascular smooth muscle cells [28], [50], [65]. This higher molecular mass species has been proposed to be the result of glycosylation or to be due to tight association of a complexing protein. Staggered start codons at the beginning of the NOX4 gene also provide the possibility of a shift in the ORF; signal sequence cleavage from the protein upon translocation represents another possible explanation.

To expand these initial results, we sought to define the region of recognition by our antibody. Plasmid constructs were created through insertion of stop codons within the ORF of an N-terminal affinity tagged NOX4 gene to divide the extracellular E-loop region into gradually excluding segments of amino acids (Supplementary Fig. 3A). Transfection into HEK293 cells resulted in protein expression of each construct, as determined by Western analysis with Myc-tag antibody. Evaluation with the NOX4 antibody demonstrated that recognition of the longest truncated construct (Stop 4) was significantly more intense than the full length NOX4 protein (Supplementary Fig. 3B). This is most likely due to greater exposure of the protein recognition region, affording better access for the NOX4 antibody to bind. Further truncation completely abolished the ability of the antibody to recognize the NOX4 protein. Therefore, the amino acids recognized by antibody 47-6 encompass those found between the sites where stop codons 3 and 4 were placed (Fig. 1C, Supplementary Fig. 3C).

With a specific recognition epitope established, further sequence-based analysis was undertaken for antibody characterization to determine if mAb 47-6 may exhibit species specific interactions, or may be broadly applicable to NOX4 identification in many eukaryotes. Taxonomic studies have established that the NOX4 enzyme and the NOX1-3 subfamilies originated from a common branch, with expression across many species, including but not limited to mouse, rat, chicken, cow, and fish [64], [66], [67]. Mouse and rat NOX4 isoforms share 98% sequence identity; sequence alignment of human, mouse, and rat NOX4 isoforms demonstrated a significant region of dissimilarity coinciding with the region of 47-6 recognition (Supplementary Fig. 4A). To interrogate whether this sequence divergence may be sufficient to prevent cross-reactivity, Western analysis was carried out using samples expressing human (MycDDK-HsNOX4) and mouse (MmNOX4-3HA6His) NOX4. As observed in Supplementary Fig. 4B, the NOX4 monoclonal antibody 47-6 only identifies the human NOX4 protein. Importantly, unlike other antibodies from academic labs developed with C-terminal NOX4 antigens, 47-6 mAb recognition is unperturbed by C-terminal affinity tagged constructs (MmNOX4-3HA6His, Supplemental Fig. 5). While it is unfortunate that the 47-6 antibody is incapable of NOX4 detection in rodent model studies and knockout systems, xenograft studies utilizing implanted human tumors in animals may be more effectively monitored with this tool, due to its inability to detect contaminating NOX4 from the carrier system.

### 3.3 NOX4 monoclonal antibody efficacy in confocal and flow cytometry studies

To further establish utility, the immunoreactivity of the NOX4 antibody was initially characterized with an overexpression system. Two stable HEK293 NOX4 clones, both verified to express the NOX4 protein, stabilize p22^phox^, and produce H_2_O_2_, were selected for confocal microscopy (Supplementary Fig. 6). Both membrane-permeable and non-permeable cells were evaluated, because NOX4 is expressed at the plasma surface affording extracellular exposure of the E-loop region. After staining with a protocol utilizing 2 μg/mL 47-6, parental and vector stable clones displayed no significant background signal, nor did cells incubated with equimolar rabbit IgG (Fig. 1D). Both overexpression clones displayed intense staining, with or without Triton-X permeabilization, consistent with an extracellular antigen derived antibody recognizing an exposed membrane protein. These results suggested that the 47-6 antibody is sensitive enough to utilize in confocal studies to verify localization, and should be further challenged with an endogenous system.

We sought to assess the applicability of our NOX4 mAb for labeling of native antigen in HEK293 NOX4 overexpressing stable cells by flow cytometry (Supplementary Fig. 7). Both NOX4 overexpressing clones demonstrate considerable NOX4 mAb bound to the permeabilized overexpressing cells compared to vector control and parental cells. Increased fluorescence was also noted with intact live overexpressing NOX4 cells; this result confirms the extracellular antigen design utilized for antibody development, and further supports localization of NOX4 at the plasma membrane surface of the HEK293 NOX4 stable overexpression cell lines.

### 3.4 Evaluation of activity perturbation by the NOX4 mAb

Sequence modifications, differences in transmembrane spanning regions, as well as extensions at the amino termini all may be critical for regulatory and functional differences between individual NADPH oxidase family members. Regarding NOX4, chimeric proteins developed by mixing portions of the phagocytic NOX2 and NOX4 enzymes revealed ROS production by NOX4 is dependent on the B loop region, perhaps through interaction with the FAD/NADPH binding domain, and further undefined structural elements in the transmembrane or extracellular regions [54]. Subsequent fluorescence polarization studies defined an activity-dependent B-loop interaction with the FAD domain of NOX4, proposed to maintain a discreet distance between the FAD region and the membrane helix bound heme moiety(s), affording facile electron transfer [68]. Further targeted investigation has also defined the E-loop region of NOX4 as essential to the ROS species produced by this enzyme [61]. A single site mutation, at His-222 (H222Q), was shown to cause a shift in ROS production, converting the NOX4 enzyme into a superoxide producer. Further mutational analysis of this loop region, through single site mutations of two cysteine residues, provided corroborating results, establishing control of H_2_O_2_ production at this loop region.

To explore the possibility that our NOX4 E-loop directed antibody might influence ROS production, both Amplex Red and L-012 assays were performed to monitor the type and amount of ROS species produced by a NOX4 overexpression system in the presence of 47-6 mAb (Fig. 1E-F, Supplementary Fig. 8). Constitutive hydrogen peroxide production observed with the NOX4 overexpressing system is unperturbed by control rabbit IgG treatment, and attenuated by treatment with the flavin dehydrogenase inhibitor diphenylene iodonium (DPI) (Fig. 1E). In comparison, no significant decrease in hydrogen peroxide production was observed following exposure to a range of antibody concentrations, suggesting that while antibody 47-6 binds to the E-loop region, this binding has little to no effect on ROS production. Further support for this observation was established by chemiluminescent L-012 assay; superoxide production was not notably stimulated by 47-6 mAb treatment; therefore, exposure to the 47-6 mAb does not mimic the effect of a single-site mutation within the E-loop region (Fig. 1F). The site of binding may be crucial, as the known His-222 mutation which results in superoxide production is ~20 amino acids from the site of 47-6 Ab binding (Fig. 1A-C). The stability of the antibody binding or epitope position also may not be as effective as direct mutation, or may suggest that this region does not confer significant structural support to the E-loop such that perturbation would result in ROS modification.

### 3.5 TGF-β1-mediated up-regulation of NOX4 in lung fibroblasts

Transforming growth factor beta1 (TGF-β1) belongs to a family of structurally related, secreted polypeptide cytokines that control cellular growth, proliferation, differentiation, and apoptosis [69]. Stimulation of smooth muscle cells [25], [70], hepatocytes [36], [71], and fibroblasts [34], [37], [72] with low concentrations of TGF-β1 has been shown to significantly increase NOX4 expression and ROS production. A myriad of effects have been related to the resultant NOX4 up-regulation, varying by cell type. In studies of fetal rat and human hepatocytes, treatment with TGF-β1 results in oxidative-stress mediated apoptosis [73], [74]. Regulated at the transcriptional level, NOX4 was established in this system as the only significantly up-regulated NOX isoform contributing directly to caspase 3 activation and Rac-independent apoptosis [71]. Human pulmonary artery smooth muscle cells have also demonstrated a TGF-β1 response, with increased NOX4 expression and ROS production facilitating proliferation through the traditional SMAD 2/3 pathway, independent of MAP kinase signaling [25]. In contrast, differentiation is supported by TGF-β1-stimulated, nuclear-localized NOX4 up-regulation, increased ROS production, and p38 phosphorylation in both murine embryo fibroblasts (NIH3T3) and human lung fibroblasts (CCL-210) [34].

To support the investigative precedent for TGF-β1 mediated up-regulation, we chose the human lung fibroblasts (CCL-210) for stimulation and Western analysis. After 24 h of starvation, fibroblasts were treated for fixed times with 2 ng/mL TGF-β1, and subsequently harvested for both RNA and protein expression evaluation (Fig. 1G). Significant up-regulation of NOX4 was noted, as early as 6 h, with maximal expression at 24–48 h. Consistent with the gene expression levels of NOX4, our monoclonal antibody detected increasing levels of NOX4 protein upon TGF-β1 treatment, with increasing treatment time resulting in higher NOX4 expression between 6 h and 24 h treatments.

### 3.6 NOX4 immunoreactivity is higher in human tumors than in normal tissue

**Fig. 2:**
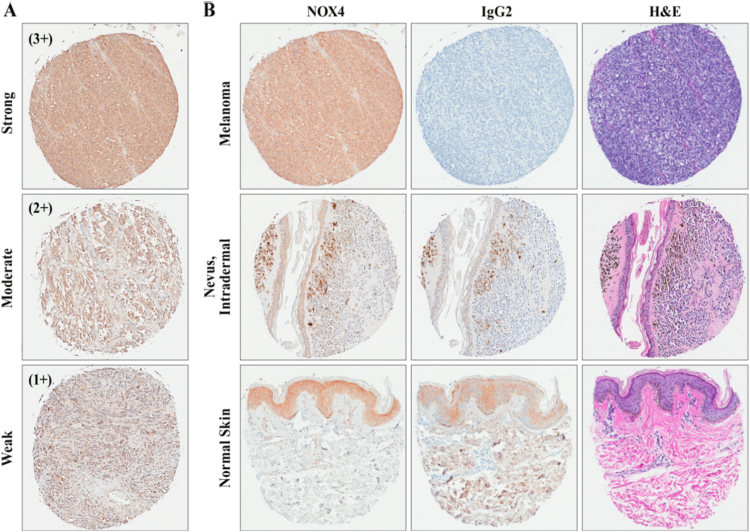
Immunohistochemical (IHC) staining of melanoma tissues demonstrating NOX4 expression. (A) Depiction of melanoma tissues across scoring intensities from weak (1+), moderate (2+), and strong (3+). (B) Tissues stained with NOX4 mAb (47-6), haemotoxylin and eosin (H&E), or normal rabbit isotype control (IgG2) across normal and malignant skin samples. All samples are digitally magnified at 10x.

Having established the ability of our monoclonal antibody to detect low levels of NOX4 in an endogenous system, we focused our studies on an immunohistochemical examination of NOX4 expression in human cancers. NOX4 expression levels, relative to uninvolved normal tissues, were assessed in a comprehensive library of formalin-fixed, paraffin-embedded tissue samples; 549 specimens of various malignancies and matched normal tissues, inflammatory and benign lesions, including samples of brain, bladder, breast, colon, esophagus, head and neck, kidney, liver, lung, lymph node, ovary, pancreas, prostate, skin, soft tissue, stomach, testis, thyroid, uterine cervix, and uterus were evaluated and scored for NOX4 expression (Supplementary Table 2). Staining intensity was assessed by a pathologist (HM) using the following scoring system: 0 (negative/no or blush staining), 1+ (weak), 2+ (moderate), and 3+ (strong) (Fig. 2A). The percentage of stained tumor/lesional cells was estimated for each case where, 0= 0% −10% were considered negative, 1 = 10–24%, 2=25–49%, 3≥50-of positive cells. Cases with moderate to strong cytoplasmic reactivity of the antibody on ≥10% of cells were considered to be high expressors.

**Fig. 3:**
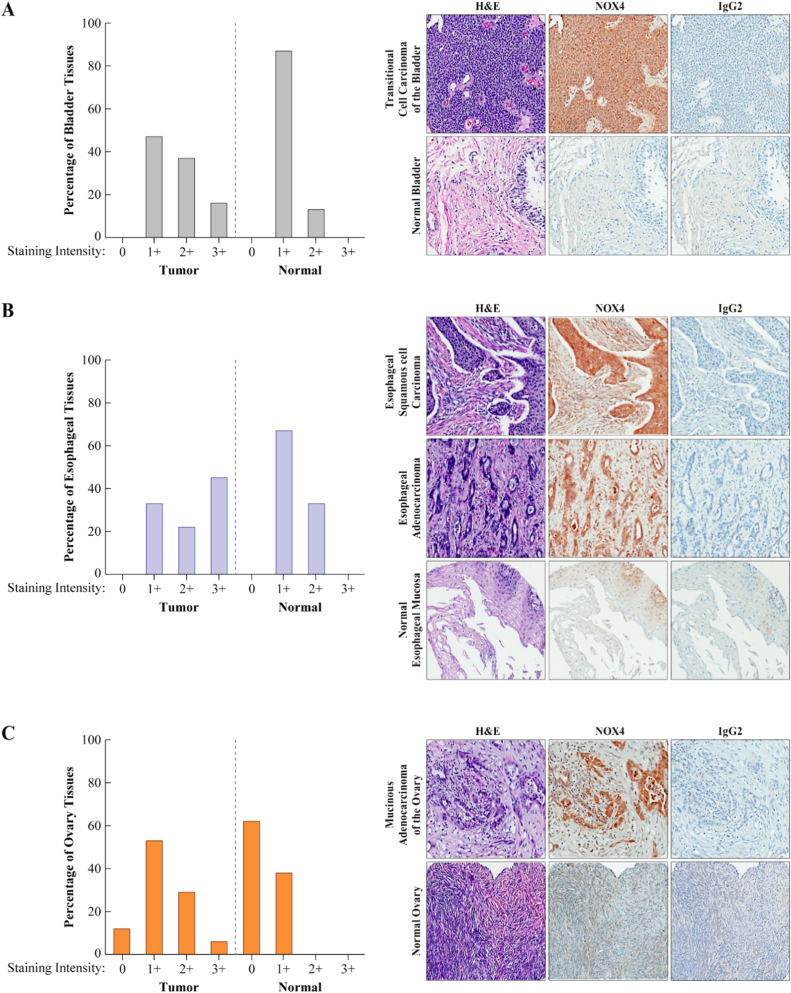
Representative examples of NOX4 immunostaining from a broad array of malignant versus normal tissues. IHC staining of BioMax Multiple Organ TMA MC6163 for NOX4 expression was scored utilizing the following system: 0 = negative/no or blush staining, 1+ = weak, 2+ = moderate, and 3+ = strong. The percentage of bladder (A), esophageal (B), and ovarian (C) tissues stained with NOX4 is illustrated graphically (left), with tissue images depicting the staining pattern of NOX4 mAb (47-6), haemotoxylin and eosin (H&E), or normal rabbit isotype control (IgG2) (right). Malignant tissue staining demonstrated a significant NOX4 expression increase relative to the corresponding normal tissues.

Differential NOX4 staining was observed in bladder, esophagus, head and neck, ovary, and prostate cancers and melanoma compared to corresponding normal tissues (Table 1, Fig. 2, Fig. 3, Supplementary Fig. 9). NOX4 positive expression was detected in 19/19 transitional cell carcinomas of the bladder; more than 50% of the samples were high positive, compared to the corresponding normal bladder epithelial tissue where the majority were low positive (87%). Similarly, esophageal carcinoma samples showed a differential expression of NOX4 antigen relative to its corresponding non-neoplastic esophageal mucosa (>75% of the adenocarcinoma and >50% of the squamous carcinoma cases were high positive). All the head and neck squamous cell carcinomas, regardless of their origin, expressed NOX4; most (approximately 80%) were high positive. Supplementary Fig. 9A showed reactivity of NOX4 antibody with squamous cell carcinoma. Normal epithelia of the tongue (lower image) was negative with the NOX4 mAb except the basal layer. Neither the normal prostate tissue nor the samples of benign prostate hyperplasia were high positive, in contrast to over 50% of the adenocarcinoma cases (Supplementary Fig. 9B).

**Table 1:** Distribution of expression levels of NOX4 in human malignancies. Tissues that were scored [+] as weak (1+) for NOX4 staining were considered low expressors, and those that stained moderate (2+) to strong (3+) high expressors; unstained/unscored tissues are related as [-]. Represented within parentheses are the relative percentages of tissues. See Supplementary Table 2 for specific details on scoring.

**Organ**	**Pathologic diagnosis**	**No. [+]/Total (%)**	**No.[-]/Total (%)**	**Low Expressors [No. (%)]**	**High Expressors [No. (%)]**
**Bladder**	Transitional cell carcinoma	19/19 (100)	0 (0)	9 (47)	10 (53)
	Normal bladder Tissue	8/8 (100)^a^	0 (0)	7 (87)	1 (13)

**Esophagus**	Adenocarcinoma	9/9 (100)	0 (0)	2 (22)	7 (78)
	Squamous cell carcinoma	9/9 (100)	0 (0)	4 (44)	5 (56)
	Normal esophagus tissue	6/6 (100)^b^	0 (0)	4 (67)	2 (33)

**Head and Neck**	Squamous cell carcinoma	19/19 (100)	0 (0)	4 (21)	15 (79)
	Normal tongue tissue	6/6 (100)^c^	0 (0)	2 (33)	4 (67)

**Ovary**	Serous adenocarcinoma	8/8 (100)	0 (0)	6 (75)	2 (25)
	Mucinous adenocarcinoma	7/9 (78)	2/9 (22)	3 (43)	4 (57)
	Normal ovary tissue	3/8 (38)	5/8 (62)	3 (100)	0 (0)

**Skin**	Squamous cell carcinoma	12/14 (86)	2/14 (14)	10 (83)	2 (17)
	Malignant melanoma	14/15 (93)	1/15 (7)	7 (50)	7 (50)
	Intradermal nevus	2/4 (50)	2/4 (50)	1 (50)	1 (50)
	Normal skin tissue	0/8(0)	8/8 (100)	0 (0)	0 (0)

**Prostate**	Adenocarcinoma	12/19 (63)	7/19 (37)	5 (42)	7 (58)
	Hyperplasia of prostate	1/2 (50)	1/2 (50)	1 (100)	0 (0)
	Normal Prostate	4/6 (67)	2/6 (33)	4 (100)	0 (0)

a Only 5/8 samples have epithelium.

b 3/6 have squamous epithelium.

c 5/6 have squamous epithelium.

**Fig. 4:**
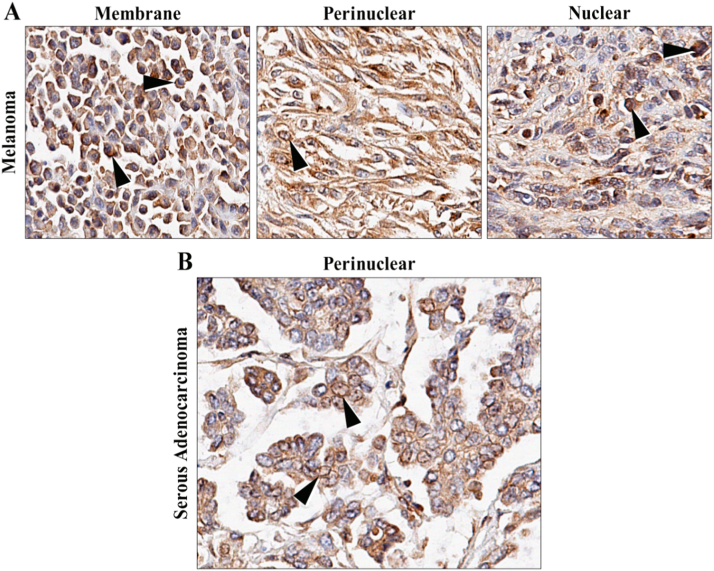
Distribution of NOX4 (47-6) immunostaining in melanoma and serous ovarian adenocarcinoma tissues. (A) Melanoma tissues from BioMax TMA MC6163 illustrate membrane (left), perinuclear in a spindle cell melanoma (center), and nuclear (right) IHC staining. Arrows highlight representative sections which define the NOX4 mAb staining distribution. (B) Serous ovarian adenocarcinoma tissue stained with 47-6 mAb demonstrated NOX4 localization in perinuclear regions. An arrow highlights one section of distinct perinuclear staining. All photomicrographs were taken at 40X digital magnification.

Of significance, the antibody to NOX4 reacted with almost all melanomas (14/15, 93%) and none of the normal skin samples (n=8) or the compound nevi (0/2) (Fig. 2B). Fifty percent of melanomas were high positive for NOX4 (7 of 14 melanomas). Most melanomas showed diffuse cytoplasmic positivity with a detectable membrane distribution in half of the cases; nuclear and perinuclear distribution was observed in 1/3 of the samples (Fig. 4A). By subtype, serous ovarian carcinomas demonstrated NOX4 overexpression in 8/8 tissues (100%) and mucinous ovarian tissue displayed moderate to strong NOX4 staining for 7/9 (78%), samples, relative to the corresponding normal ovarian tissues 3/8. Most of the high positive samples were histologically categorized as moderately- to well-differentiated. Fig. 4B, shows a sample from a moderately differentiated serous ovarian adenocarcinoma that demonstrated perinuclear localization of NOX4.

NOX4 was also detected in a significant proportion of small cell lung carcinomas (11/12, 92%), gastric carcinomas (12/17, 71%) and breast carcinomas (18/20, 90%). Uterine (cervix) squamous cell carcinomas had the lowest expression of positive tumors (56%). This is the first broad, systematic immunohistochemical analysis of NOX4 expression in human malignancies. Previous studies have examined small subsets of cancers, including bladder [17], breast and ovarian [75], and liver tumors [18]. We have identified several malignant histologies (esophageal, head and neck, and prostate carcinomas) that have not previously been reported to show substantial NOX4 expression by IHC. Further investigation is necessary to determine the relevance of NOX4 activity in these unexplored systems.

### 3.7 Investigation of NOX4 in ovarian cancer cell lines

The accumulation of reactive oxygen species during the progression of ovarian cancer [76], [77], [78], [79], [80], [81], and its effects on increased MKP3 degradation and subsequent ERK 1/2 activation [82] or the promotion of tumor angiogenesis [83] have been documented, but few studies have investigated NOX4 as a hydrogen peroxide source contributing to the development or progression of ovarian malignancies [22], [84].

**Fig. 5:**
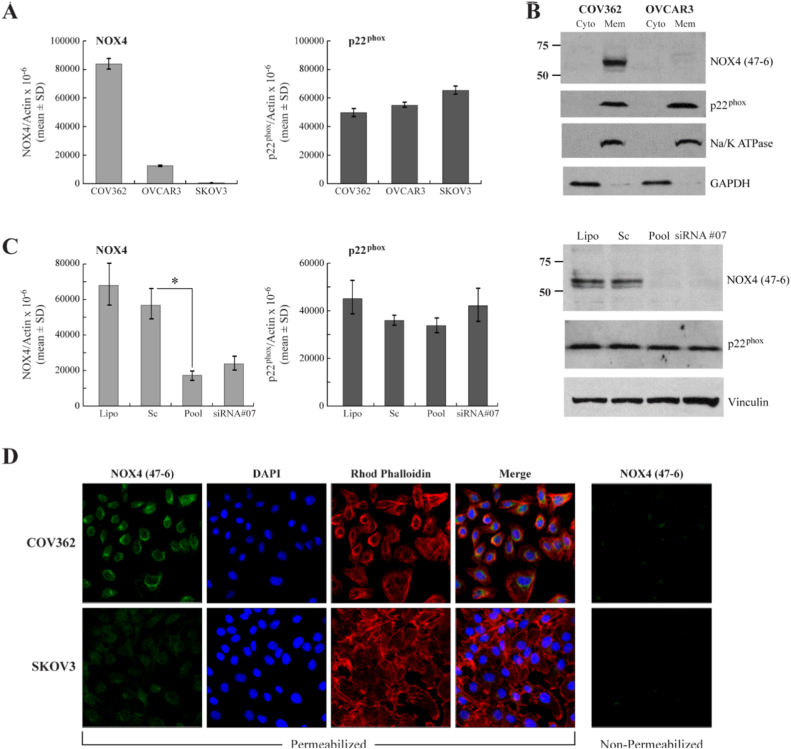
Endogenous NOX4 levels observed with NOX4 mAb exposure. (A) qPCR evaluation of NOX4 levels in COV362, SKOV3, and OVCAR3 ovarian cancer cell lines. (B) Membranes isolated from COV362 and OVCAR3 cells were evaluated by Western analysis against cytosolic fractions; 60 μg total protein loaded per lane. (C) Transient knockdown of the NOX4 protein in COV362 cells; comparison of lipofectamine only (Lipo) and non-targeting siRNA (Sc) controls to NOX4 siRNA pool (Pool) and NOX4 specific siRNA #07 treated cells, evaluated on the RNA (left) and protein level (right). *, *p* < 0.05. D) Confocal microscopy of COV362 and SKOV3 cells, staining for the NOX4 enzyme, the nucleus (DAPI), and cytoskeleton (Rhodamine Phalloidin) under permeabilized and non-permeabilized conditions (NOX4 47-6 only, right).

Supported by the results of the tissue microarray analysis and previous observations of NOX4 expression in cell lines profiled by the genomic database from the cancer cell line encyclopedia (CCLE) [4], several ovarian cancer cell lines were examined for levels of NOX4 and p22^phox^, the necessary components for enzymatic hydrogen peroxide production. COV362, OVCAR3, and SKOV3 cell lines were evaluated by quantitative PCR to determine the expression level of each gene (Fig. 5A). Significant levels of NOX4 were found in COV362, seven times greater than in OVCAR3 cells. Western analysis of total membrane preparations of these cell lines with the mAb 47-6 demonstrated this disparity at the protein level (Fig. 5B). To confirm that our antibody successfully recognized the NOX4 protein, we employed short interfering RNA (pooled NOX4 siRNA and specific siRNA #07) to knockdown endogenous NOX4 in COV362 cells (Fig. 5C). As expected, the NOX4 knockdown achieved was verified both at the RNA and protein level, leaving the level of p22^phox^ unperturbed.

Having verified the presence of NOX4 protein in the COV362 cell line, we utilized confocal microscopy to define cellular localization. COV362 and SKOV3 cells, chosen to afford the broadest difference in NOX4 expression levels, were evaluated both under permeabilized and unpermeabilized conditions (Fig. 5D). No significant staining was noted at the cellular surface of non-permeable cells, suggesting little to no expression of NOX4 at the plasma membrane. Interestingly, COV362 cells demonstrated perinuclear staining in the membrane-permeable system, defined by NOX4-related green fluorescence hugging the DAPI stained nuclei while remaining easily distinguishable from cytoskeletal staining (rhodamine phalloidin). Similar staining is absent in the SKOV3 cells, further supporting the identity of NOX4. Localization within multiple intracellular structures cannot be ruled out, as regions of focused, increased staining were noted in the perinuclear region, reminiscent of the endoplasmic reticulum (ER) or Golgi apparatus. Recent studies support NOX4 intracellular localization (nucleus, nuclear envelope, mitochondria, ER), as opposed to plasma membrane association, when observed in endogenous systems [30], [49], [51], [52].

## 4 Discussion

Efforts continue to clarify the role of hydrogen peroxide in intracellular signaling cascades responsible for induction and maintenance of an oncogenic phenotype in cancer cells [85], [86]. NOX4 is among many ROS producing enzymes under study as potential sources of ROS producing oxidative DNA damage [87], [88]. Unfortunately, high quality, commercial monoclonal antibodies against NOX4 have not been available to aid these studies [60]. The few available academic antibody sources, while reliable, were not designed for extracellular binding and/or because they are polyclonal probes, suffer from prominent non-specific binding and variation in quality from multiple animal hosts. To address this need, we developed a novel NOX4 monoclonal antibody whose antigen resides in the extracellular E-loop region of the enzyme and that has been validated for immunohistochemical studies.

Our NOX4 antibody (47-6) was raised in a rabbit host, with a dual antigen inoculation strategy using peptide and fixed overexpressing NOX4 cells. This new tool was validated for isoform specificity (Fig. 1B), through detection of overexpressing protein without displaying significant non-specific bands. Further characterization, by Western analysis of truncated E-loop constructs was undertaken, defining a 21 amino acid region recognized by the 47-6 antibody (Fig. 1C, Supplementary Fig. 3). This sequence displays a lack of fidelity with both mouse and rat NOX4 isoforms, unlike the remainder of the protein where high sequence conservation is observed (Supplementary Fig. 4A). Comparison of overexpressed NOX4 constructs confirmed the inability of our monoclonal antibody to bind to mouse NOX4 protein (Supplementary Fig. 4B). The incapability of our 47-6 antibody to detect mouse or rat NOX4 may prove opportune in the context of implanted human tumor studies, providing the ability to isolate NOX4 enzymatic changes from host contamination. To move beyond overexpression systems, TGF-β1 mediated up-regulation of NOX4 was employed to demonstrate antibody sensitivity. TGF-β1 has been identified as a stimulant of NOX4 expression in several cell systems, including smooth muscle cells, hepatocytes, and fibroblasts; CCL-210 lung fibroblasts treated with 2 ng/mL TGF-β1 up-regulated NOX4 as shown by qPCR and detected through Western analysis with 47-6 (Fig. 1G).

Therapeutic interventions focusing on ROS formation can involve strategies to either increase or decrease oxidative stress [2]. Increased ROS scavenging, by enhanced expression of antioxidants (SOD, catalase) or decreased pro-oxidant enzyme expression, may decrease oxidative tone and depress tumor growth and migration [89], [90], [91]. Alternatively, disrupting oxidant removal, causing an accumulation of excess ROS in malignant cells, could initiate a beneficial apoptotic cascade [92]. NOX4 oxidant production appears to be related to the structural integrity of the E-loop region [61]. Our E-loop recognizing monoclonal antibody was therefore tested for effects on both hydrogen peroxide and superoxide production (Fig. 1E-F, Supplementary Fig. 8). Amplex Red and L-012 substrates were utilized as probes for hydrogen peroxide and superoxide production, respectively. Neither assay, monitored for up to 90 min, revealed a significant effect on ROS production when NOX4 overexpressing HEK293 cells were incubated with the 47-6 antibody. Although it is unfortunate that our antibody does not appear to hold therapeutic value as a direct enzymatic effector, the importance of the E-loop region toward enzymatic function should continue to be explored.

Application of our antibody to a human tumor tissue microarrays has provided novel insights into the expression of NOX4 in human tumors (Fig. 2, Fig. 3, Table 1, Supplementary Table 2). High levels of NOX4 expression were found in previously-characterized tissues, including kidney, brain, and pancreas; however, significant differential expression, defined by greater expression in malignant tissues versus normal, was noted only in bladder, esophageal, head and neck, ovarian and prostate carcinomas and malignant melanoma. No major studies, to date, have focused primarily on NOX4 in esophageal adenocarcinoma or squamous cell carcinoma. This observation may provide additional relevance to a recent study suggesting that TGF-β−related oxidative stress promoted pre-malignant basal cell hyperplasia in the esophagus which was associated with a 2.5-fold increase in NOX4 mRNA [93]. Our IHC data have identified esophageal carcinoma as a potential focus for future NOX4 research, joining ongoing but preliminary efforts in bladder, head and neck, and ovarian cancers, and malignant melanoma.

Ovarian cancer has the highest morality rate of all gynecological cancers and is the fifth leading cause of cancer-related death in women [94], [95]; and oxidative stress has been linked to the pathogenesis of ovarian cancer [76], [79], [81], [96]. It is possible that NOX4 may play an underappreciated role in ovarian cancer, an hypothesis supported both by observations of increased NOX4 expression in malignant ovarian tissues and through cell line profiling (Fig. 3, Fig. 5) [7]. Our monoclonal antibody confirmed that COV362 ovarian cancer cells clearly demonstrate expression of NOX4 enzyme; expression of its necessary activity partner p22^phox^ was also validated. NOX4 expression, through confocal microscopy, was specifically shown to be localized to the nuclear region, without significant plasma membrane association (Fig. 5D). Unchecked intracellular hydrogen peroxide generation from NOX4 could foster DNA damage and promote aberrant cellular signaling; further investigative efforts are required to establish the role of NOX4 in this disease. The lack of plasma membrane localized NOX4, furthermore, suggests that efforts to evaluate ROS generation in tumor biology should focus on tools that measure intracellular ROS, including fluorescent dyes, and DNA damage markers (i.e. anti 8-oxoguanine antibodies). By relying on assay strategies that measure extracellular hydrogen peroxide production, studies may be missing crucial information regarding the effects of NOX4-dependent ROS generation.

In summary, we have developed a novel immunologic tool for study of the NOX4 enzyme. Our monoclonal antibody (47-6) has proven to be isoform selective and sensitive for investigative efforts that involve endogenous expression levels, and therefore is an important resource to support future NOX4 studies. Insights into the role of NOX4 in tumor biology, provided by IHC, demonstrate marked overexpression of NOX4 in several malignancies, several for the first time. Our future studies will focus on understanding the specific role of H_2_O_2_ in oxidative signaling and DNA oxidation for tumors that have been shown to overexpress NOX4.

## Author disclosure statement

No competing financial interests exist.
