# Supplementary material for: Decoding NADPH oxidase 4 expression in human tumors
Source: Redox Biol. 2017 May 26;13:182–95. doi: 10.1016/j.redox.2017.05.016 (PMC5458090; doi:10.1016/j.redox.2017.05.016)
Supplement: Supplementary file 1 — Supplementary material [file mmc1.docx]

**Supplementary Figure 1**

Validation of multiclones by Western analysis prior to subcloning. ELISA screening of rabbit hybridoma multiclones with peptide immunogen identified 30 positive clones. Western blot analysis with supernatants (1:5 dilution in 5% milk/TBST) identified 6 multiclones (#1, 33, 34, 35, 47 and 71) capable of detecting the NOX4 protein from NOX4 overexpression versus vector control lysates. ELISA values and western blot sections are shown for each multiclone selected for subcloning.


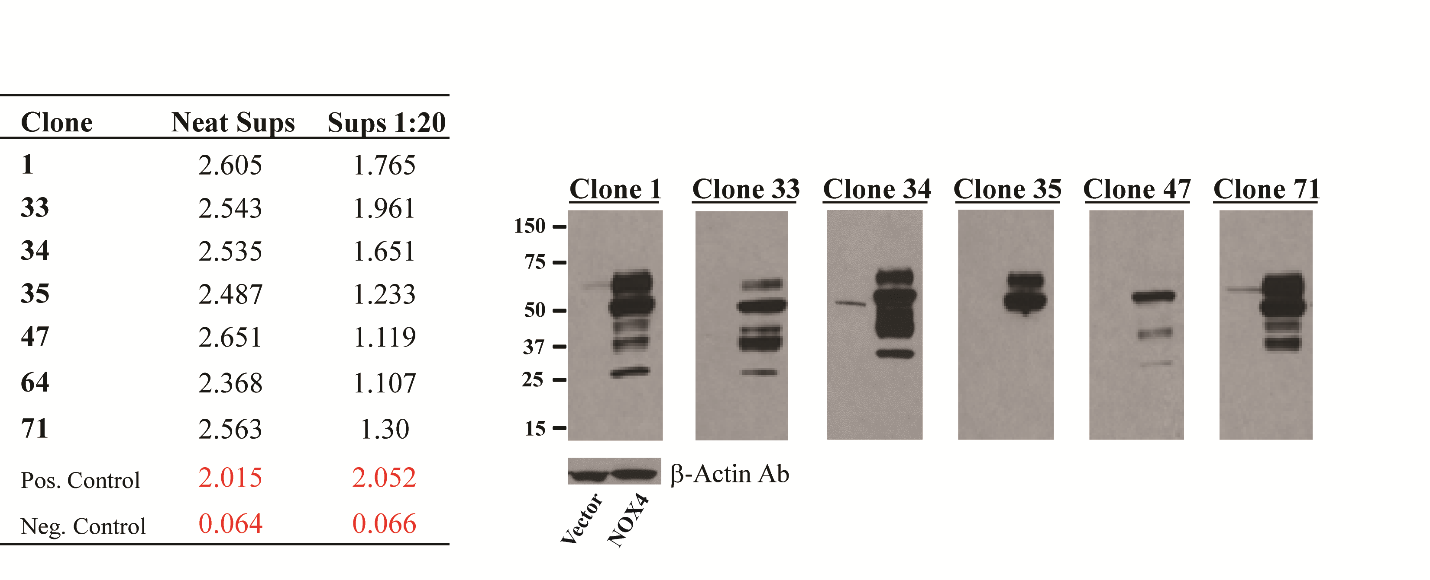


**Supplementary Figure 2**

Sequencing results of the purified NOX4 antibody obtained from rabbit hybridoma clone 47-6. A) The consensus sequence obtained from the heavy chain variable region (V_H_). B) The consensus sequence obtained from the light chain variable region (V_L_).


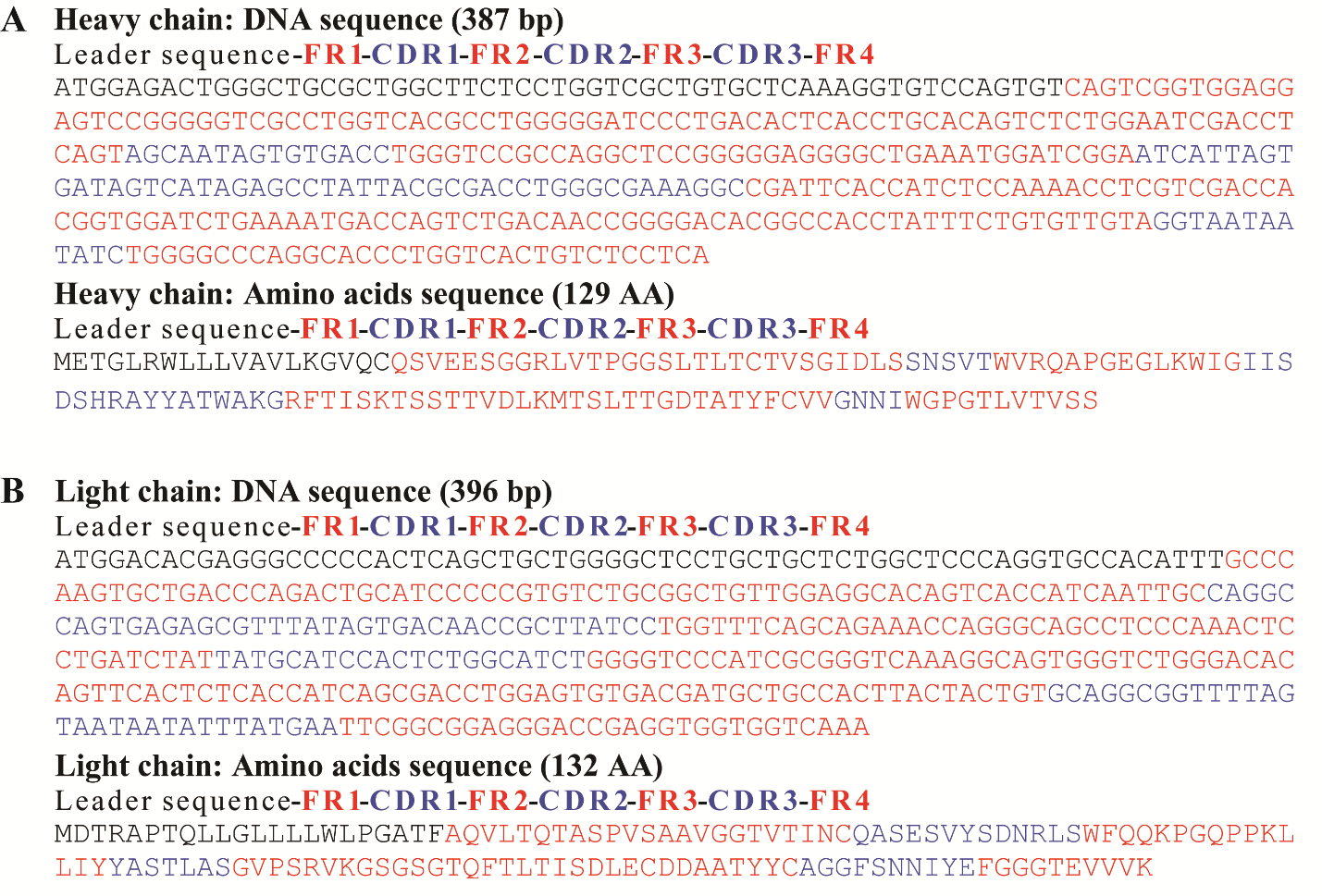


**Supplementary Figure 3**

Verification of NOX4 E-loop site recognition. A) Schematic illustration of pCMV-MycDDK-HsNOX4 (FL) and the deletion constructs (Stop 1-4) generated to evaluate the mAb 47-6 site of recognition. The N-terminal Myc-tag (triangle), E-loop (gold) and FAD/NADPH binding domain (BD, rectangle) are highlighted. B) Western analysis of full length MycDDK-HsNOX4 (FL, 69.7 kDa) and deletion constructs Stop1 (26.6 kDa), Stop 2 (28.3 kDa), Stop 3 (30.1 kDa) and Stop 4 (32.6 kDa) after transient transfection into HEK293 (3 μg cDNA, 48h). Eighty micrograms total protein was loaded per lane. C) Schematic illustration of the NOX4 E-loop; the validated recognition sequence is highlighted (red) and stop codon placements for truncated constructs noted.


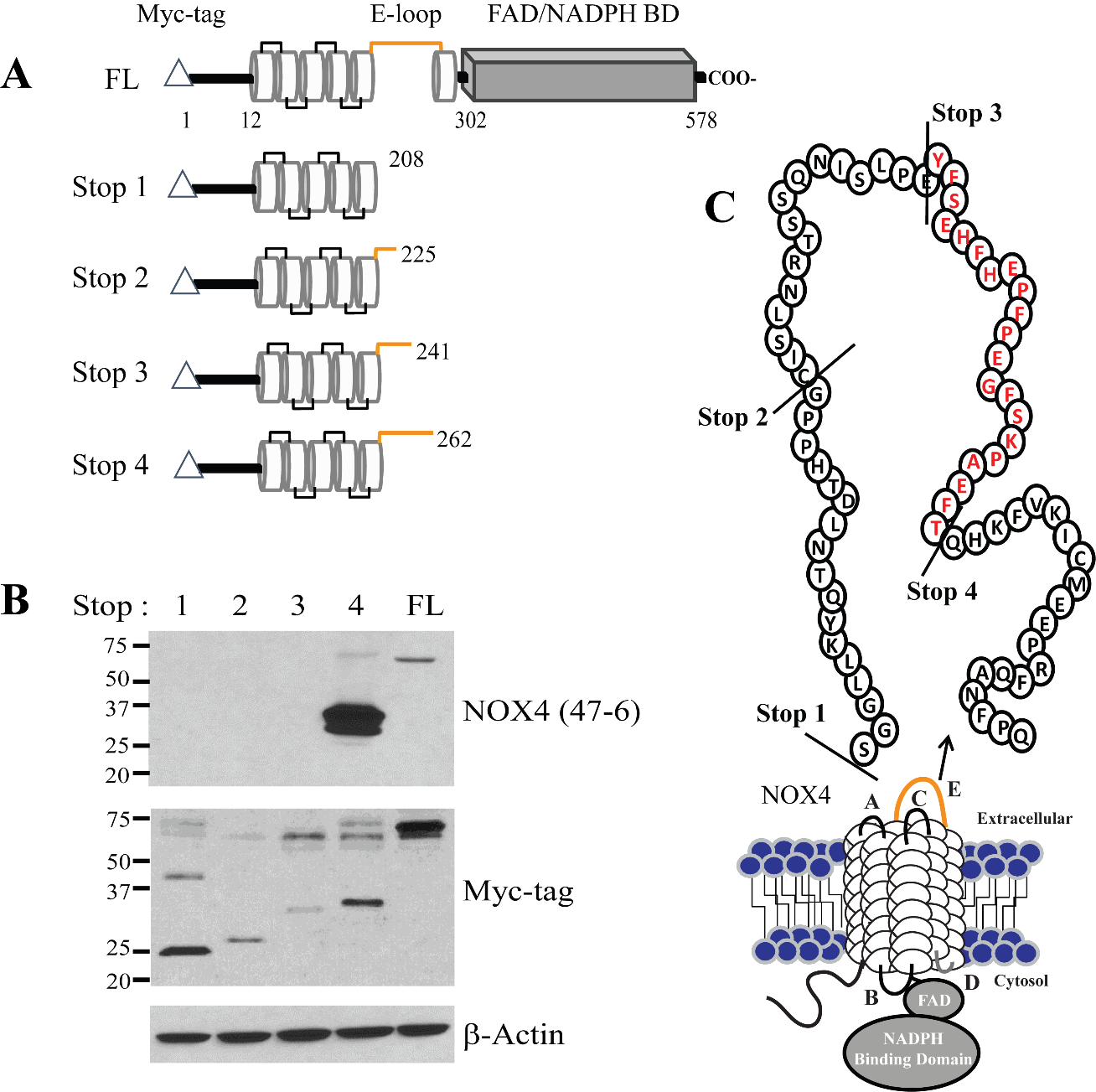


**Supplementary Figure 4**

A) Multiple sequence alignment performed using Clustal Omega of mouse (Mm, *M. musculus*), rat (Rn, *R. norvegicus*) and human (Hs, *H. sapiens* isoform A) NOX4. The 47-6 NOX4 antibody recognition sequence is highlighted in red. B) Quantitative RNA and Western analysis is shown for HEK293 cells transfected with human or mouse NOX4 cDNA (4 μg, 48h). HEK293 cells transiently transfected with empty vector (Con), human MycDDK-NOX4 cDNA (Hs) or mouse NOX4-3xHA6His cDNA (Mm) were verified by total RNA isolation and loaded against control cell lysates from MycDDK-NOX4 (Myc) and HA-NOX5 (HA) stable cell lines. 90 μg HEK293 transfected protein and 20 μg stable clone control proteins were loaded per lane.

**
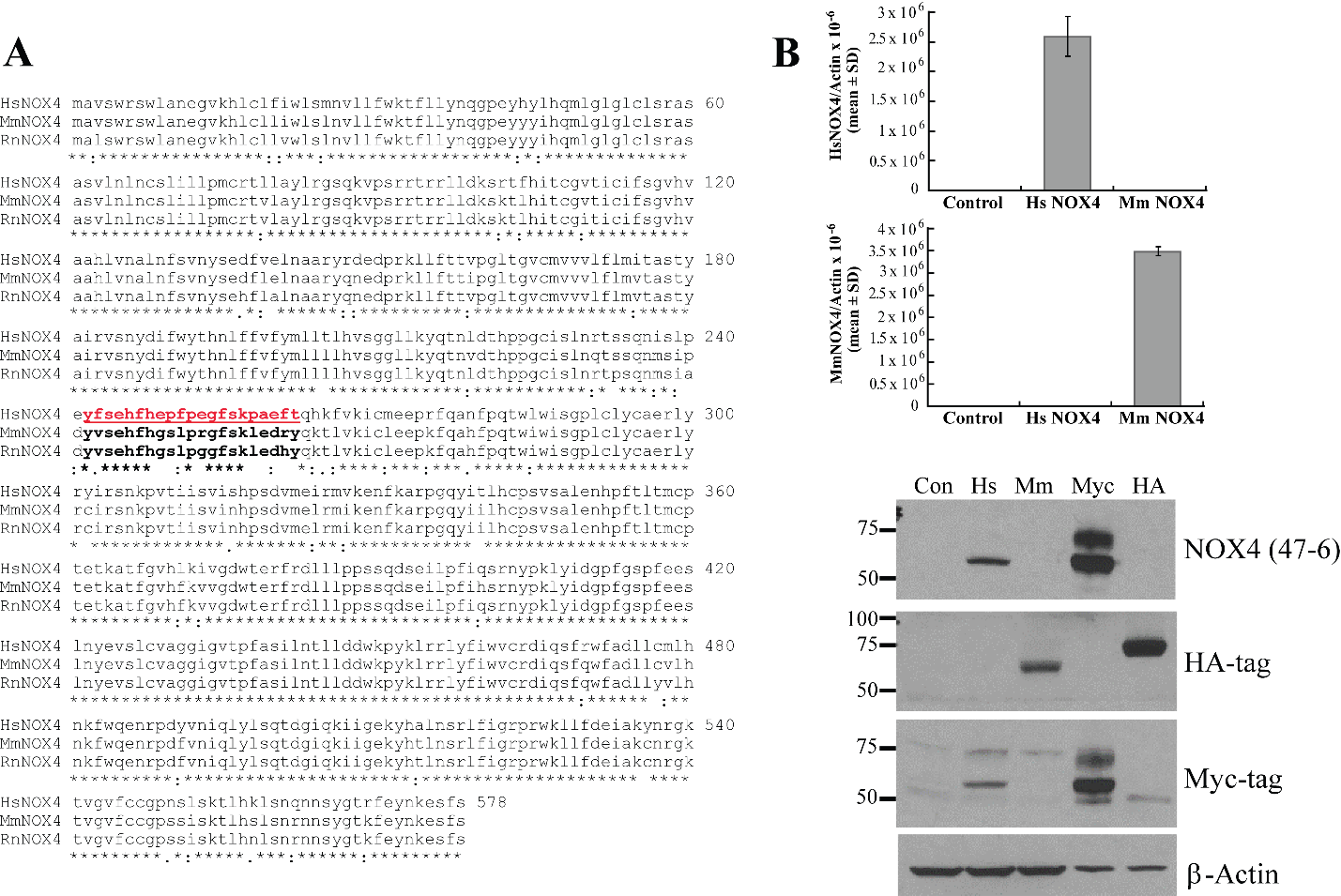
**

**Supplementary Figure 5**

Demonstration of affinity tag effect on NOX4 Ab recognition. Dürr and Shah Lab NOX4 antibodies were developed against C-terminal regions of the NOX4 protein (Table 1). A) Evaluation of transiently transfected human (Hs) and mouse (Mm) NOX4 cDNAs (4 μg, 48 h) in HEK293 cells against empty vector (Con) for N-terminally tagged constructs pCMV-MycDDK-HsNOX4 and pCMV-6His-MmNOX4) and B) C-terminally tagged mouse NOX4 (pCMV-MmNOX4-3HA-6His) provided an interesting divergence in recognition (90 μg total protein/lane). Stable empty vector (Vec) and MycDDK-HsNOX4 (Myc) clones confirm recognition by all NOX4 antibodies (20 μg total protein/lane). The Shah Lab NOX4 polyclonal antibody significantly recognizes mouse NOX4, with weaker human isoform affinity. The Dürr Lab NOX4 monoclonal antibody recognizes both human and mouse isoforms, when affinity tags are located at the N-terminus (Panel A), however a C-terminal affinity tag clearly perturbs recognition (Panel B).


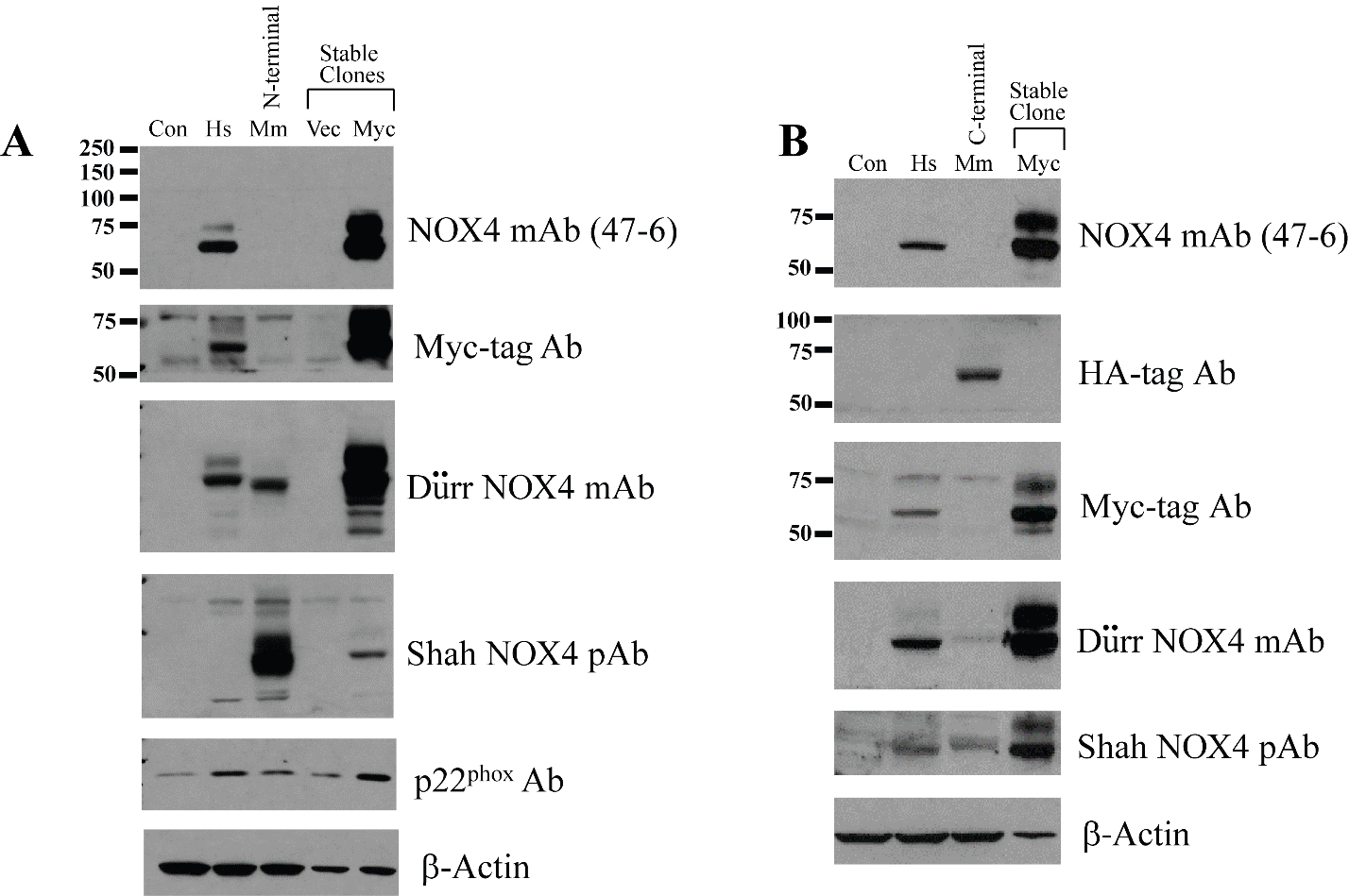


**Supplementary Figure 6**

Evaluation of stable clones to validate functional NOX4 incorporation. RNA (top), western blot (middle) and H_2_O_2_ production (bottom) is shown for HEK293 parental (HEK), vector control (Vec), and MycDDK-NOX4 stable clones 1 (WT1) and 2 (WT2).

**
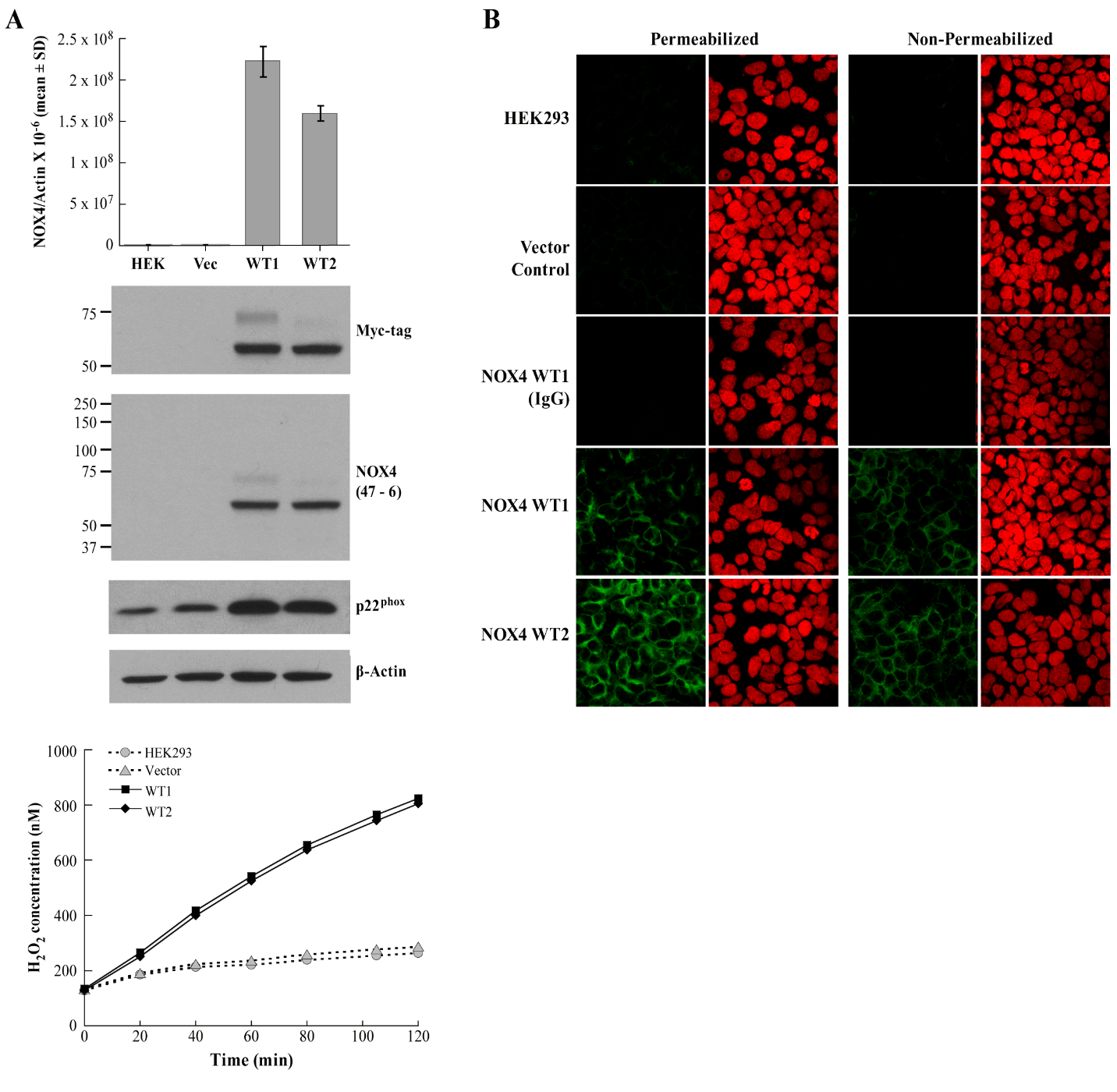
**

**Supplementary Figure 7**

Flow cytometry study of overexpressed NOX4 protein with mAb 47-6. NOX4 protein detection in permeabilized (A) and intact (B) HEK293 stable overexpression NOX4 cell lines (WT1 and WT2) compared to parental and vector control cells was evaluated after rabbit IgG and 47-6 mAb labeling; fluorescence detection was achieved after Alexa Fluor 488 goat anti-rabbit secondary staining.

**
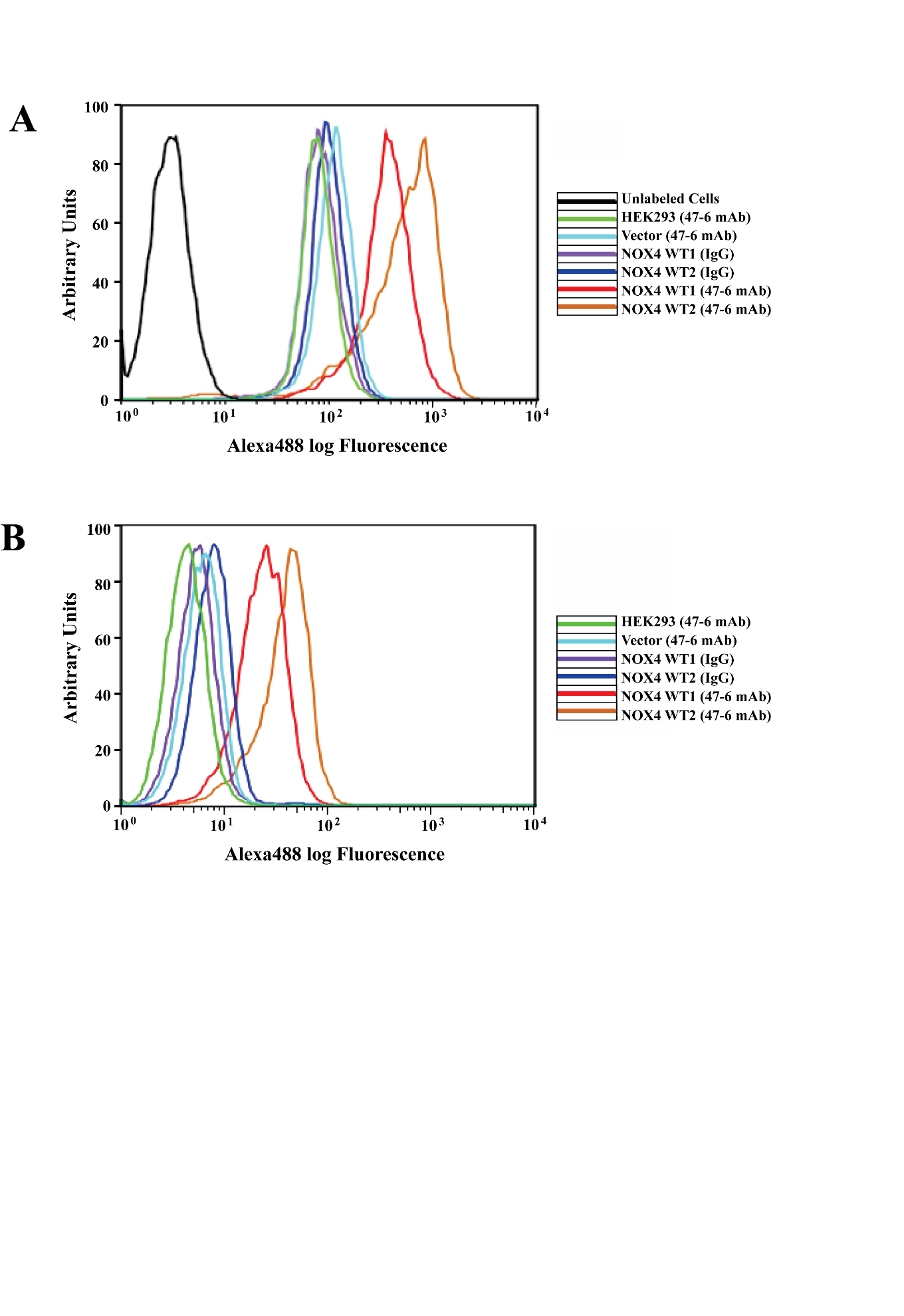
**

**Supplementary Figure 8**

Negligible effect observed for mAb 47-6 on the activity of the NOX4 enzyme. A) Amplex Red assay measuring H_2_O_2_ production by HEK293 NOX4 stable clone 1 versus stable vector control cells. NOX4 overexpressing stable cells were pre-incubated for 30 min at 37 ^o^C with 47-6 mAb, rabbit IgG or DPI before addition to Amplex reagent solution. The assay 90 min time point is compared across all samples. B) L-012 Assay was used to measure and compare superoxide generation across superoxide producing stable mutant H222Q NOX4 and WT NOX4 overexpressing stable cells treated with 47-6 mAb or rabbit IgG. SOD (4U) treatment was used to verify superoxide production; data at the 30 min time point is compared across all samples.


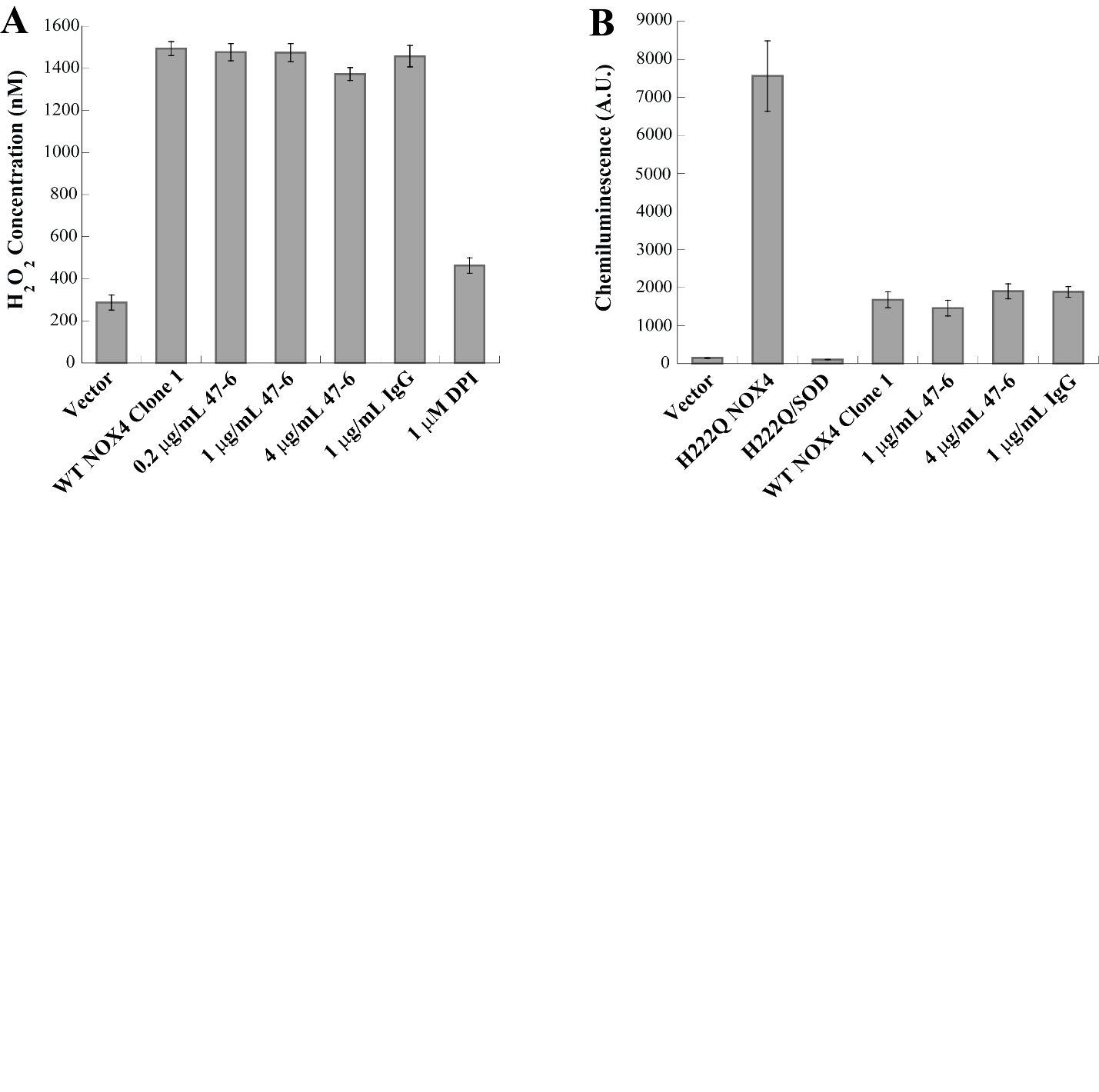


**Supplementary Figure 9**

Patterns of expression of NOX4 in human multitumor microarray MC6163 tissues. A) Head and Neck, Squamous cell carcinoma and normal tongue tissues. B) Prostate adenocarcinoma and normal tissues.

**
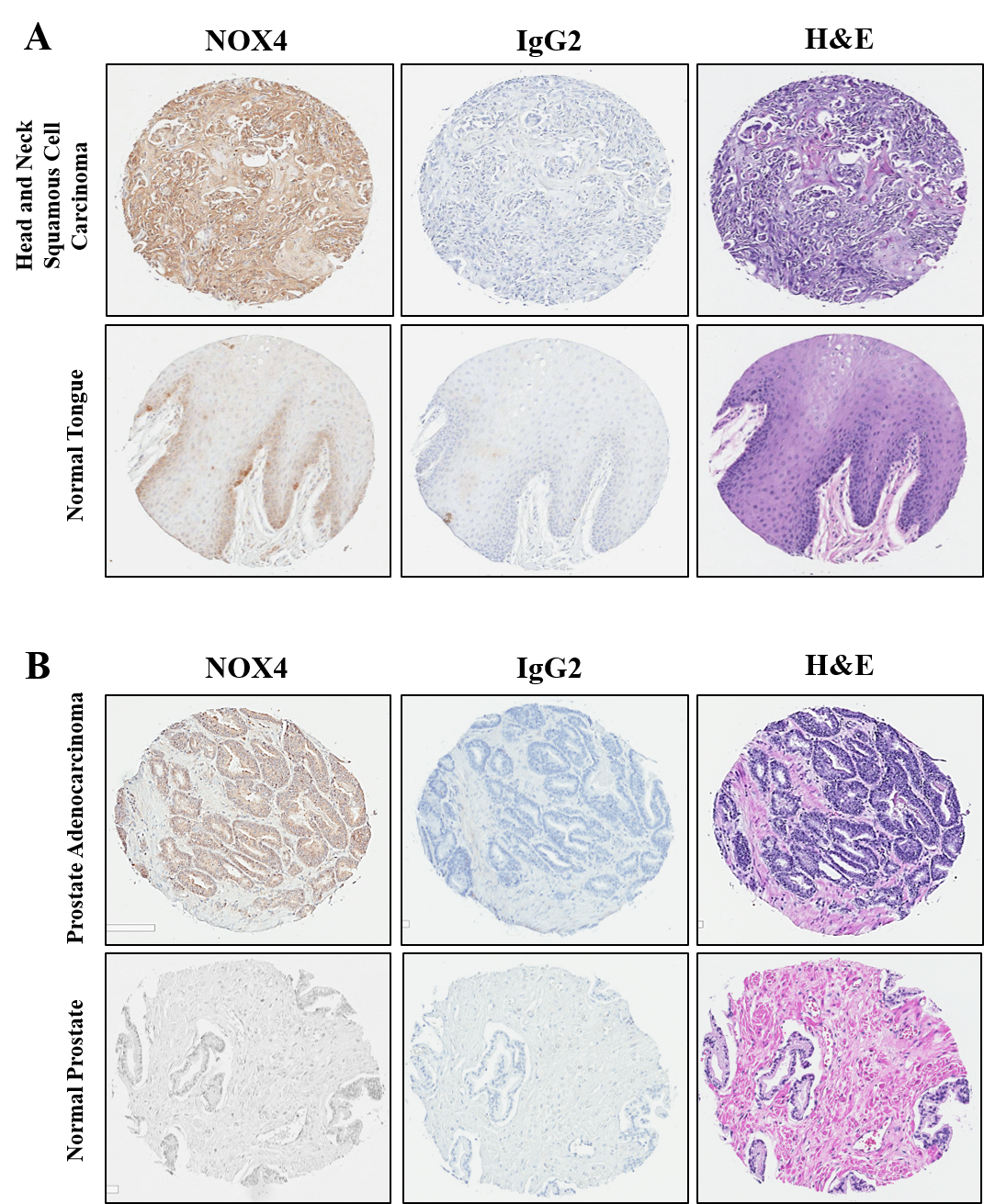
**

**Supplementary Table 1**

| **Quikchange primers utilized for stop codon insertions in the ORF of NOX4 in the pCMV-MycDDK-NOX4 vector** | |
| --- | --- |
| **NOX4 stop 1 1711 a** | 5’- gacgttgcatgttTGAGGAGGGCTGC-3’ |
| **NOX4 stop 1 1711 b** | 5’- GCAGCCCTCCTCAaacatgcaacgtc-3’ |
| **NOX4 stop 2 1763 a** | 5’- CCCTCCCGGCTGaATCAGTCTTAACCG-3’ |
| **NOX4 stop 2 1763 b** | 5’- CGGTTAAGACTGATtCAGCCGGGAGGG-3’ |
| **NOX4 stop 3 1811 a** | 5’- CCTTACCAGAGTAgTTCTCAGAAC-3’ |
| **NOX4 stop 3 1811 b** | 5’- GTTCTGAGAAcTACTCTGGTAAGG-3’ |
| **NOX4 stop 4 1874 a** | 5’- CCGGCAGAGTTTACCtAGCACAAATTTGTG-3’ |
| **NOX4 stop 4 1874 b** | 5’- CACAAATTTGTGCTaGGTAAACTCTGCCGG-3’ |

**Supplementary Table 2** Results of NOX4 mAb staining of multiple organ TMA (MC6163).

| **Organ** | **Tumor Type/Pathologic Diagnosis** | **Subtype** | **No. [+]/Total (%)** | **No.[-]/Total (%)** | **Low Expressors (WeAk) No. (%)** | **High Expressors (Moderate+strong) No.(%)** | **Staining Pattern†** |
| --- | --- | --- | --- | --- | --- | --- | --- |
|  |  |  |  |  |  |  |  |
|  |  |  |  |  |  |  |  |
| **Bladder** | Carcinoma | Transitional cell carcinoma | 19/19 (100) | 0 (0) | 9 (47) | 10 (53) | C +++, M+, F++, E++, I++ |
|  | Normal bladder tissue |  | 8/8 (100) only 5/8 have epithelium | 0 (0) | 7 (87) | 1 (13) | C ++, E+++, F+++, I++, SM+ |
| **Cerebrum** | Astrocytic tumors | Astrocytoma | 10/10 | 0 (0) | 2 (20) | 8 (80) | C+++, E++, I+++ |
|  |  | Glioblastoma multiforme | 10/10 (100) | 0 (0) | 2 (20) | 8 (80) | C+++, E++, I+++ |
|  | Normal cerebrum tissue |  | 8/8 (100) | 0 (0) | 1 (12) | 7 (88) | C+++, E, NF |
| **Breast** | Carcinoma | Invasive ductal carcinoma | 18/20 (90) | 2/20 (10) | 2 (11) | 16 (89) | C+++ |
|  | Normal breast tissue |  | 8/8 (100) | 0 (0) | 1 (13) | 7 (87) | C +, E+++, F+++, I++ |
| **Colon** | Carcinoma | Adenocarcinoma | 12/12 (100) | 0 (0) | 0 (0) | 12 (100) | C+++, brush border |
|  |  | Mucinous adenocarcinoma | 7/7 (100) | 0 (0) | 1 (14) | 6 (86) | C++ |
|  | Normal colon tissue |  | 8/8 (100), only 4/8 have epithelium | 0 (0) | 0 (0) | 8 (100) | C+++, F++, E+++, I++ |
| **Esophagus** | Carcinoma | Adenocarcinoma | 9/9 (100) | 0 (0) | 2 (22) | 7 (78) | C+++ |
|  |  | Squamous cell carcinoma | 9/9 (100) | 0 (0) | 4 (44) | 5 (56) | C+++ |
|  | Normal esophagus tissue |  | 6/6 (100); 3/6 have squamous epithelium | 0 (0) | 4 (67) | 2 (33) | C++ (basal layer); E++ |
| **Head and neck** | Carcinoma | Squamous cell carcinoma | 19/19 (100) | 0 (0) | 4 (21) | 15 (79) | C+++, I+++, F++, E++ |
|  | Normal tongue tissue |  | 6/6 (100); 5/6 have squamous epithelium | 0 (0) | 2 (33) | 4 (67) | C+++ (basal layer), E++, SM+ |
| **Kidney** | Carcinoma | Clear cell carcinoma | 19/20 (95) | 1/20 (5) | 5 (26) | 14 (74) | C+++, M+ (in clear cells), I++, E+++ |
|  | Normal kidney tissue |  | 8/8 (100) | 0 (0) | 0 (0) | 8 (100) | C++ Luminal border of tubules.,Glomerular cytoplasmic, E +++, I+ |
| **Liver** | Carcinoma | Hepatocellular carcinoma | 18/20 (90) | 2/20 (10) | 5 (27) | 13 (73) | C+++, M+ in clear cells), KC+++, I++, E+++, BD |
|  | Normal hepatic tissue |  | 6/6 (100) | 0 (0) | 2 (33) | 4 (67) | HC ++ (lipofuscin), KC+++, E++, |
| **Lung** | Carcinoma | Squamous cell carcinoma | 5/11 (45) | 6/11 (55) | 5 (100) | 0 (0) | C+++, I+++, E++, F+ |
|  |  | Adenocarcinoma | 6/10 (60) | 4/10 (40) | 4 (67) | 2 (33) | C+++, I+++, E+, F++ |
|  |  | Large cell carcinoma | 7/11 (64) | 4/11 (36) | 4 (57) | 3 (43) | C++, I++, E+ |
|  |  | Small cell carcinoma | 11/12 (92) | 1/12 (8) | 4 (36) | 7 (64) | C+++, I++ |
|  | Normal lung tissue |  | 3/7 (43) | 4/7 (57) | 0 (0) | 3 (100) | C+ (alveolar cells), I+ |
| **Lymph node** | Lymphoma | Hodgkin's lymphoma (Mixed cellularity) | 9/9 (100) | 0 (0) | 3 (33) | 6 (67) | C+++ |
|  |  | Diffuse small B-cell lymphoma | 9/9 (100) | 0 (0) | 2 (22) | 7 (78) | C+++, I++ |
|  | Normal lymph node tissue |  | 6/7 (86) | 1/7 (14) | 0 (0) | 6 (100) | C+++ (Lymphocytes), E++ |
| **Ovary** | Carcinoma | Serous adenocarcinoma | 8/8 (100) | 0 (0) | 6 (75) | 2 (25) | C+++, stroma +, I+, E+, |
|  |  | Mucinous adenocarcinoma | 7/9 (78) | 2/9 (22) | 3 (43) | 4 (57) | C+, I+,E+, |
|  | Normal ovary tissue |  | 3/8 (38) | 5/8 (62) | 3 (100) | 0 (0) | C++ epithelium,stroma ++ |
| **Pancreas** | Carcinoma | Ductal adenocarcinoma | 13/16 (81) | 3/16 (19) | 7 (54) | 6 (46) | C+++, F++ (stroma), I++ |
|  | Normal pancreas tissue |  | 8/8 (100) | 0 (0) | 0 (0) | 8 (100) | C+++ |
| **Prostate** | Carcinoma | Adenocarcinoma | 12/19 (63) | 7/19 (37) | 5 (42) | 7 (58) | C+++ |
|  | Benign | Hyperplasia of prostate | 1/2 (50) | 1/2 (50) | 1 (100) | 0 (0) | C++ (epithelium), stroma negative |
|  | Normal prostate tissue |  | 4/6 (67) | 2/6 (33) | 4 (100) | 0 (0) | C+++ (epithelium), stroma negative |
| **Skin** | Nonmelanoma skin cancer | Squamous cell carcinoma | 12/14 (86) | 2/14 (14) | 10 (83) | 2 (17) | C++ |
|  | Melanoma | Malignant melanoma | 14/15 (93) | 1/15 (7) | 7 (50) | 7 (50) | C+++; M+, pigments |
|  | Benign melanocytic tumor | Intradermal nevus | 2/4 (50) | 2/4 (50) | 1 (50) | 1 (50) | C++, pigments |
|  | Normal skin tissue |  | 0/8(0) | 8/8 (100) | 0 (0) | 0 (0) |  |
| **Soft tissue** | Sarcoma | Fibrosarcoma | 6/7 (86) | 1/7 (14) | 5 (83) | 1 (17) | C++ |
|  |  | Neurofibrosarcoma | 0/1 (0) | 1/1 (100) | 0 (0) | 0 (0) |  |
|  |  | Well-differentiated liposarcoma | 3/4 (75) | 1/4 (25) | 1 (33) | 2 (67) | C++ |
|  |  | Myxoliposarcoma | 4/5 (80) | 1/5 (20) | 3 (75) | 1 (25) | C++ |
|  | Normal skeletal muscle tissue |  | 1/7 (14) | 6/7 (86) | 0 (0) | 1 (100) | C+, E+++ |
| **Stomach** | Carcinoma | Adenocarcinoma | 12/16 (75) | 4/16 (25) | 10 (83) | 2 (17) | C+++ |
|  |  | Undifferentiated carcinoma | 0/1 (0) | 1/1 (100) | 0 (0) | 0 (0) | C+++ |
|  | Normal stomach tissue |  | 8/8 (100) | 0 (0) | 1 (12) | 7 (88) | C+++ (epithelium), secretion |
| **Testis** | Germ cell tumor | Seminoma | 8/9 (89) | 1/9 (11) | 4 (50) | 4 (50) | C+++ |
|  | Carcinoma | Embryonal carcinoma | 6/7 (86) | 1/7 (14) | 5 (83) | 1 (17) | C+++ |
|  | Normal testis tissue |  | 7/7 (100) | 0 (0) | 4 (57) | 3 (43) | C+++ |
| **Thyroid gland** | Carcinoma | Papillary adenocarcinoma | 8/10 (80) | 2/10 (20) | 3 (37) | 5 (63) | C+++ |
|  |  | Follicular adenocarcinoma | 5/10 (50) | 5/10 (50) | 4 (80) | 1 (20) | C+++ |
|  | Normal thyroid gland tissue |  | 4/8 (50) | 4/8 (50) | 1 (25) | 3 (75) | C++, colloid positive |
| **Uterine cervix** | Carcinoma | Squamous cell carcinoma | 10/18 (56) | 8/18 (44) | 8 (80) | 2 (20) | C+++, I++ |
|  | Normal cervical canals tissue |  | 2/2 (100) | 0 (0) | 1 (50) | 1 (50) | C++, E ++, Stroma ++ |
| **Uterus** | Carcinoma | Endometrioid adenocarcinoma | 12/17 (71) | 5/17 (29) | 6 (50) | 6 (50) | C+++ |
|  | Normal endometrium tissue |  | 6/6 (100) | 0 (0) | 2 (33) | 4 (67) | C+++ |

† **+Rare; ++ Few; +++ Many; (C=Cytoplasmic; N=Nuclear; M=Membranous, E=Endothelial; Inflammatory cells =I; Fibroblasts=F; Smooth muscle cells=SM; Kupffer cells=KC; Bile ducts=BD; Hepatocytes=HC; Neurofilaments=NF)**
